# Deep convolutional networks do not classify based on global object shape

**DOI:** 10.1371/journal.pcbi.1006613

**Published:** 2018-12-07

**Authors:** Nicholas Baker, Hongjing Lu, Gennady Erlikhman, Philip J. Kellman

**Affiliations:** 1 Department of Psychology, University of California, Los Angeles, Los Angeles, California, United States of America; 2 University of Nevada, Reno, Nevada, United States of America; Technische Universitat Chemnitz, GERMANY

## Abstract

Deep convolutional networks (DCNNs) are achieving previously unseen performance in object classification, raising questions about whether DCNNs operate similarly to human vision. In biological vision, shape is arguably the most important cue for recognition. We tested the role of shape information in DCNNs trained to recognize objects. In Experiment 1, we presented a trained DCNN with object silhouettes that preserved overall shape but were filled with surface texture taken from other objects. Shape cues appeared to play some role in the classification of artifacts, but little or none for animals. In Experiments 2–4, DCNNs showed no ability to classify glass figurines or outlines but correctly classified some silhouettes. Aspects of these results led us to hypothesize that DCNNs do not distinguish object’s bounding contours from other edges, and that DCNNs access some local shape features, but not global shape. In Experiment 5, we tested this hypothesis with displays that preserved local features but disrupted global shape, and vice versa. With disrupted global shape, which reduced human accuracy to 28%, DCNNs gave the same classification labels as with ordinary shapes. Conversely, local contour changes eliminated accurate DCNN classification but caused no difficulty for human observers. These results provide evidence that DCNNs have access to some local shape information in the form of local edge relations, but they have no access to global object shapes.

## Author summary

“Deep learning” systems–specifically, deep convolutional neural networks (DCNNs)–have recently achieved near human levels of performance in object recognition tasks. It has been suggested that the processing in these systems may model or explain object perception abilities in biological vision. For humans, shape is the most important cue for recognizing objects. We tested whether deep convolutional neural networks trained to recognize objects make use of object shape. Our findings indicate that other cues, such as surface texture, play a larger role in deep network classification than in human recognition. Most crucially, we show that deep learning systems have no sensitivity to the overall shape of an object. Whereas deep learning systems can access some local shape features, such as local orientation relations, they are not sensitive to the arrangement of these edge features or global shape in general, and they do not appear to distinguish bounding contours of objects from other edge information. These findings show a crucial divergence between artificial visual systems and biological visual processes.

## Introduction

Machine vision is one of the most challenging problems in artificial intelligence. Task-general image understanding is so difficult that it constitutes an “AI complete” problem [1], that is, a problem of sufficient difficulty and generality that it requires intelligence on a par with humans. If solved, it would be considered equivalent to the first successful completion of a Turing test [2,3]. While the general problem of image understanding is still far outside the capabilities of modern artificial systems, algorithms are beginning to reach near human capabilities on certain specialized tasks. In particular, deep convolutional neural network (DCNN) algorithms are achieving previously unseen performance on object recognition tasks.

Since their first entrance [4] in the ImageNet Large Scale Visual Recognition Challenge (ILSVRC), deep convolutional networks have substantially outperformed other state of the art recognition algorithms (e.g., [5]), to the point of the practical extinction of the latter. Modern ILSVRC competitions (including 1.2 million images associated with 1000 categories) almost exclusively feature deep convolutional networks, and their error rates have continuously fallen with more powerful hardware and more sophisticated engineering. The current winner has a top-five error rate of less than 3% on the image classification task, meaning that it fails to include the correct category out of 1000 object categories in its top five most likely choices less than 3% of the time, which is even lower than human performance on the same task (~5.1%).

The impressive performance of DCNNs on natural image recognition tasks and certain apparent similarities between human physiology and the architecture of these networks suggest the natural question of whether these systems explain the capabilities of human perception and acquire similar representations to those used by the human visual system. As DCNNs approach human performance in object recognition tasks, we may ask whether or in what ways their architecture and processing mirror that of human vision. In this paper, we take up this question with special focus on object shape. In a series of five experiments, we probe the capabilities of DCNNs and humans to cope with object classifications with the goal of finding out whether trained networks overtly or implicitly encode object shape and use it to perform classifications.

To anticipate our results: Deep learning networks lack shape representations and processing capabilities that form the primary bases of human object classification. Deep learning networks do have access to some relations of local orientations that may be considered local shape constituents, but they do not appear to form global shape representations. We also show that deep learning networks make no special use of the bounding contours of objects, which most reliably define shape in human and biological vision.

### Background

DCNNs have attracted considerable attention, and several different approaches have been used to compare their performance to human object processing. Some of the similarities begin with the basic architecture. Deep convolutional neural networks perform a series of nonlinear transformations on input data such as an image in the case of object recognition. The final transformation outputs a vector of category probability values, one for each object category. Critically, early layers of these networks are not fully connected as in classical neural networks. Instead, they have convolutional windows that preserve spatial information in the image [6]. In modern DCNNs, early layers tend to operate on very local regions of the image, while deeper into the network, each node receives input from filters over a larger area of the image, allowing the network to access relations between more distant regions [4]. This network architecture has some obvious similarities with biological vision. Convolutional layers are analogous to receptive fields in visual cortex, which likewise consider more disparate regions together at higher levels of extrastriate cortex [7].

Do the similarities between DCNNs and biological vision go deeper than this basic architectural feature? One way to evaluate this is by comparing physiological activity of neural units in biological systems with the activity of certain nodes in an artificial network. Pospisil, Pasupathy, and Bair presented AlexNet, a groundbreaking DCNN, with shape stimuli to which V4 cells are optimally tuned [8] and found that there is some resemblance between node responses in intermediate layers of AlexNet and cell responses in V4, although the network response was quite sparse compared to biological systems, with many units responding to none or very few of the shape stimuli [9]. Other studies have looked at the correlation between a network’s classification accuracy and the similarity between network representations and representations in the inferotemporal gyrus (IT). Randomly varying parameters across several networks, they found that the activity of nodes in networks that perform better on the object classification task give better predictions about the activity of clusters of neurons in IT when primates are presented with the same image [10,11].

Comparisons have also been made between a network’s performance and human behavior in similar tasks. In a sense, all performance measures on image classification are a comparison to human vision, as accuracy is being measured based on labels assigned by humans [12]. However, to evaluate similarities and differences between DCNNs and human vision, it can be instructive to examine network performance on tasks for which they were not explicitly trained. Several experiments have found similarities between convolutional networks and humans in such tasks. One study used features from a convolutional network to predict the memorability of certain object segments. Features extracted from the DCNN were predictive of objects’ memorability for human subjects, suggesting that humans and networks might be attending to similar features when viewing an object [13]. In another study, Peterson, Abbott, and Griffiths found a strong correlation between similarity judgments made by DCNNs with human similarity ratings [14].

**Fig 1:**
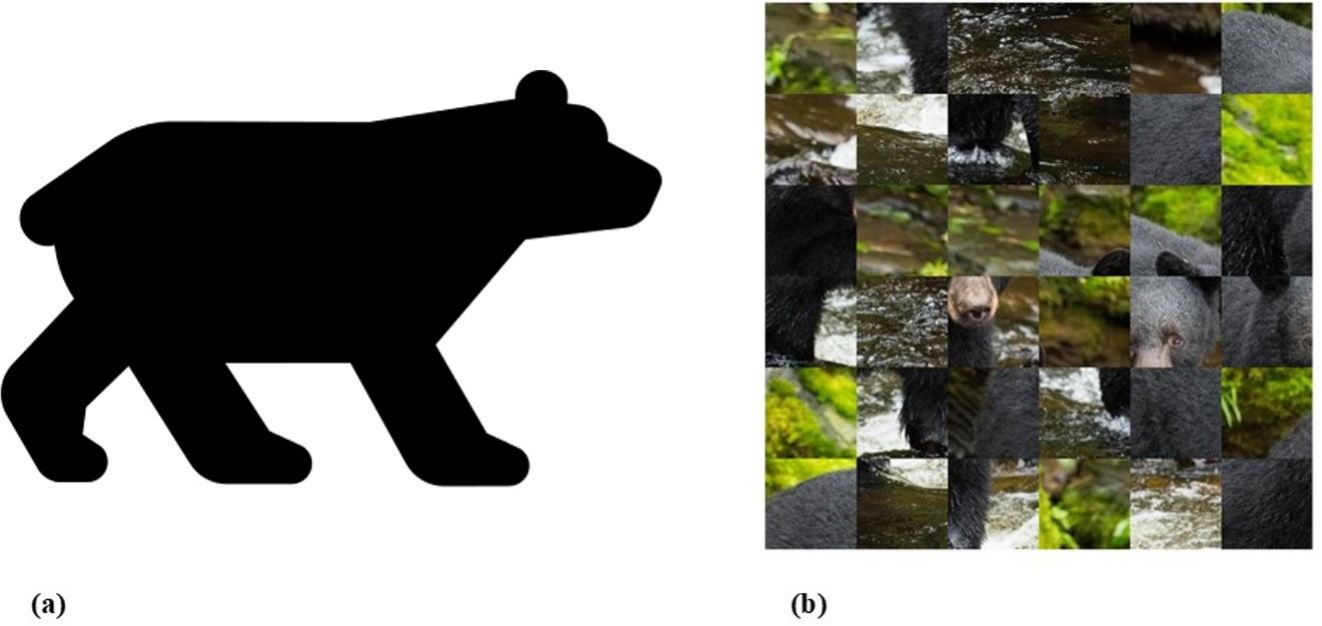
Demonstration of the importance of global shape in object recognition. (a) Silhouette of a bear; (b) Scrambled natural image of a bear (See text). Image URLs are in S2 File.

Although it is interesting to observe similar performance level for object recognition between DCNNs and biological vision, it is unclear whether the systems process information for object recognition in a similar manner. The present paper focuses on the perception of shapes, the most important cue for object recognition [15]. Objects can be recognized accurately despite impoverishments across every other visual dimension provided that global shape information is preserved [16, 17]. For example, consider the image pair presented in Fig 1. In Fig 1A, the information available for recognition has been significantly reduced across several feature dimensions. The object has no texture or background context, and the information along its contour has been simplified. Still, it is far more easily recognized as a bear than the object in Fig 1B, where cues like texture and context are preserved, but object shape is interrupted. Similarly, an ordinary line drawing, or even a few well-chosen lines as in a Picasso sketch, readily allows object recognition via shape perception processes in humans.

If deep networks are to be taken as models of human perception, we would expect object shape to be a critical component of their classification decisions. Currently, it is unclear to what extent shape representations play a role in object recognition in DCNNs. Kubilius, Bracci, and Op de Beeck conducted several intriguing experiments that suggest deep networks do have shape representations that are reasonably similar to human shape representations [18]. Networks were able to classify object silhouettes with ~40% accuracy and had some sensitivity to non-accidental features of an object, which are thought to be important for recognition in human observers [16]. They also compared the impact of shape cues on recognition performance for the networks with different architectures (e.g., different number of layers) and found some evidence that deeper networks did better on tasks where object shape was important to performance.

On the other hand, some research on DCNNs is difficult to reconcile with the claim that they utilize global shape of objects in detecting and recognizing objects. Szegedy, Zaremba, Sutskever, Bruna, Erhan, Goodfellow, and Fergus found that perturbation of a small subset of image pixels could result in consistent misclassification of the image, across multiple DCNNs, despite the changes being undetectable to human observers [19]. In the perturbed images, global shape of the object is unchanged, so the change in classification revealed that the global shape information of objects is likely not used for recognizing objects in the DCNNs. Another study used evolutionary algorithms to develop images that networks classified as certain objects with a high degree of confidence, despite a total absence of object shape in the images [20]. Zhu, Xie, and Yuille tested DCNN classification accuracy for images in which the object to be classified was removed [21]. They found that despite the object not actually being present in the image, networks performed reliably better than chance on the classification task, based purely on contextual information. These examples suggest that shape might be neither sufficient nor necessary for recognition in DCNNs.

More systematic tests are needed to understand whether or not, or in what ways, DCNNs process object shape. The fact that a network’s responses are sensitive to variables other than shape (such as texture or color) may reflect a valid use of information in its training history. The supervised learning method with which DCNNs are trained is agnostic about what information to consider when classifying an image. In natural images, texture and shape information are often highly correlated, so we can learn little about what cue is most relevant to the network’s classification without disentangling them. Reduction of performance by disruption of other variables may indicate that shape representations do not predominate, but such outcomes do not necessarily imply that shape information is not captured or potentially usable within the network. We term this “the latency problem”. To show that shape information is not implicitly captured or usable in a system, more systematic tests are required than simply showing that variables other than shape can be decisive in classification performance. Shape information may nevertheless be something DCNNs can in principle capture, but it may be latent in the system, overshadowed by other informational variables relevant for classification. Conversely, tests that have suggested that DCNN’s do use shape information have not distinguished local shape features from more global shape characteristics, nor have they disentangled responses to local orientation relations in visible contours from the more shape-defining contributions of the bounding contours of objects.

Questions about shape processing in DCNNs can be considered at three different levels: First, do deep networks trained to recognize objects use shape features in their classification decision? Second, can deep networks be trained to use shape information in their classification decisions? Third, can formal analysis of deep networks’ computational processes tell us what kinds of shape features they can and cannot extract from an image? In the current study, we focus on the first question, aiming to understand the capabilities that deep networks automatically learn through training on natural images. We consider the study of trained networks to be particularly important for two reasons. First, the attention garnered by DCNNs relates to the success of trained networks in object classification tasks. Both for theoretical understanding of these achievements and for practical applications, understanding how DCNNs achieve their high classification is important. Second, comparisons between biological vision systems and DCNNs require understanding how trained networks are operating, and require an understanding of the role of shape processing in particular. A sizable literature has recently emerged finding similarity between DCNNs trained on object recognition tasks and human perception [9–11, 13, 14, 22–26]. These efforts highlight the importance of understanding the functioning of trained DCNNs that are successful in object recognition.

In the experiments reported here, we presented DCNNs trained for object recognition (and, where appropriate, human observers) with stimuli intended to reveal information about the usability of global shape and bounding contours. The series of experiments was designed to provide multiple sources of information with regard to the latency problem in characterizing shape capabilities, in the process clarifying the roles of texture information and object shape in deep convolutional networks’ classification decisions. To carry out these studies, we tested two commonly used deep convolutional networks: AlexNet [4] and VGG-19 [27]. AlexNet has eight layers and is the deep network that started the DCNN revolution for object recognition. VGG-19 is deeper, 19 layers, and approaches the state of the art in object classification. Our approach was to use a variety of systematically modified stimuli to reveal the contribution of shape information to network responses. In Experiment 1, we examined the relative importance of overall shape and texture information by using objects in which the overall shape was preserved, but different texture, from another object, was superimposed on the object’s silhouette. In Experiments 2–4, we tested the networks on images with impoverished or altered texture and context information by using glass figurines, object outlines, and silhouettes. Following up the results and hypotheses emerging from these experiments, we tested the networks on images with manipulations that altered shape at a global level, while largely preserving local shape features, and vice versa (Experiment 5).

## Results

### Experiment 1

As noted earlier, shape is of great importance in human perception of objects, and shape information predominates in human object recognition. Prior tests of DCNNs have yielded some evidence that they classify by means of shape, whereas other work revealed examples in which images with textural similarity, but no shape in common with an object, were classified as that object with a high degree of confidence [20]. In Experiment 1, we directly compared convolutional networks’ use of shape and texture information in their classification decision. Using object silhouettes with no surface information, we overlaid a texture from a different object on top of the black figural region. We then compared the networks’ preference for both the object whose shape is displayed and the object whose texture is displayed.

#### Experiment 1 Method

**Fig 2:**
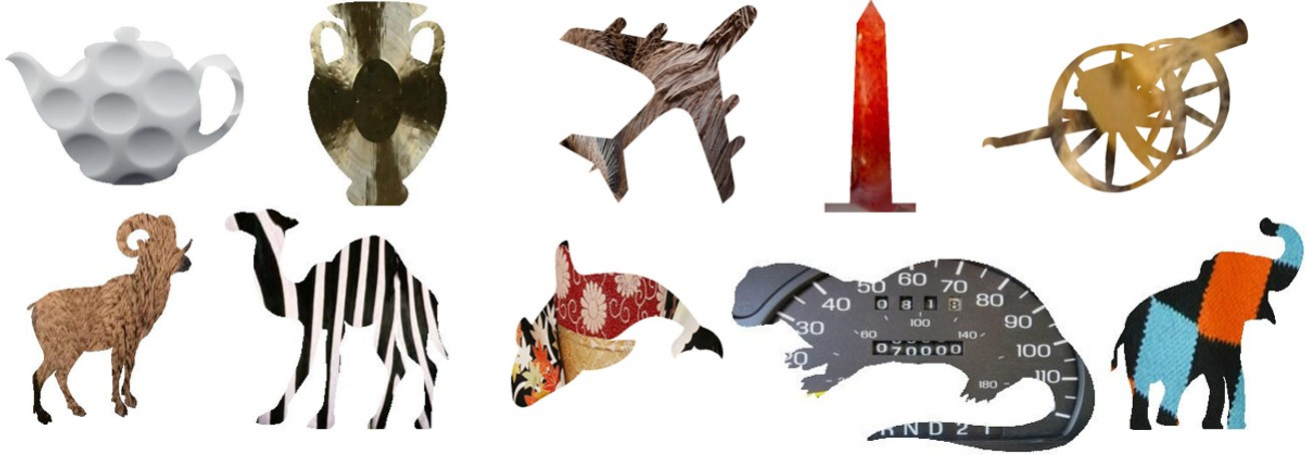
Sample stimuli used in Experiment 1. The bounding shape of an object was combined with the texture of a different object to generate each image. **a)** Shape: Teapot | Texture: Golf ball; **b)** Shape: Vase | Texture: Gong; **c)** Shape: Airplane | Texture: Otter; **d)** Shape: Obelisk | Texture: Lobster; **e)** Shape: Cannon | Texture: Pineapple; **f)** Shape: Ram | Texture: Bison; **g)** Shape: Camel | Texture: Zebra; **h)** Shape: Orca | Texture: Kimono; **i)** Shape: Otter | Texture: Speedometer; **j)** Shape: Elephant | Texture: Sock. The full image set is displayed in Figs 3–6.

*Test stimuli*. Forty images of object silhouettes and 40 natural images were obtained from internet sources (for URLs of original images, see S2 File). Half of the object silhouettes were animals, and half manmade artifacts. In the ImageNet database, about 40% of categories are animals, and 50% are manmade objects (the last 10% are other inanimate objects like food). For each silhouette, a texture from one of the natural images was overlaid on the object. All shapes and textures were taken from the 1000 object categories on which the network was trained. See Fig 2 for examples.

***Network*.** Tests were conducted on a pre-trained VGG-19 network.

#### Experiment 1 results

**Fig 3:**
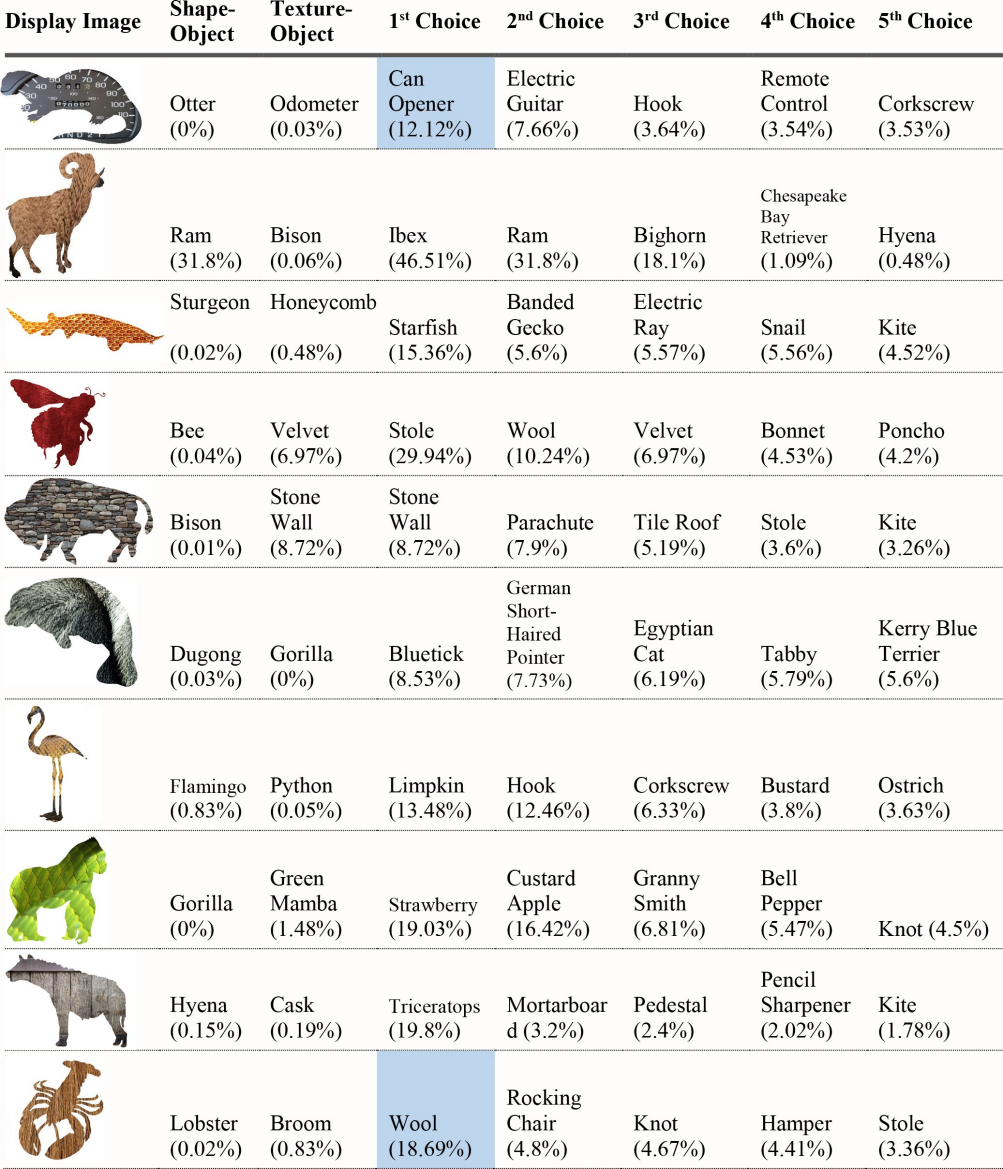
Network classifications for the stimuli presented in Experiment 1 Part 1. The left most column shows the image presented. The second column in each row names the object from which the shape was sampled. The third column names the object from which the texture silhouette was obtained. Probabilities assigned to the object name in columns 2 and 3 are shown as percents below the object label. The remaining five columns show the probabilities (as percents) produced by the network for its top five classifications, ordered left to right in terms of probability. Correct shape classifications in the top five are shaded in blue and correct texture classifications are shaded in orange.

**Fig 4:**
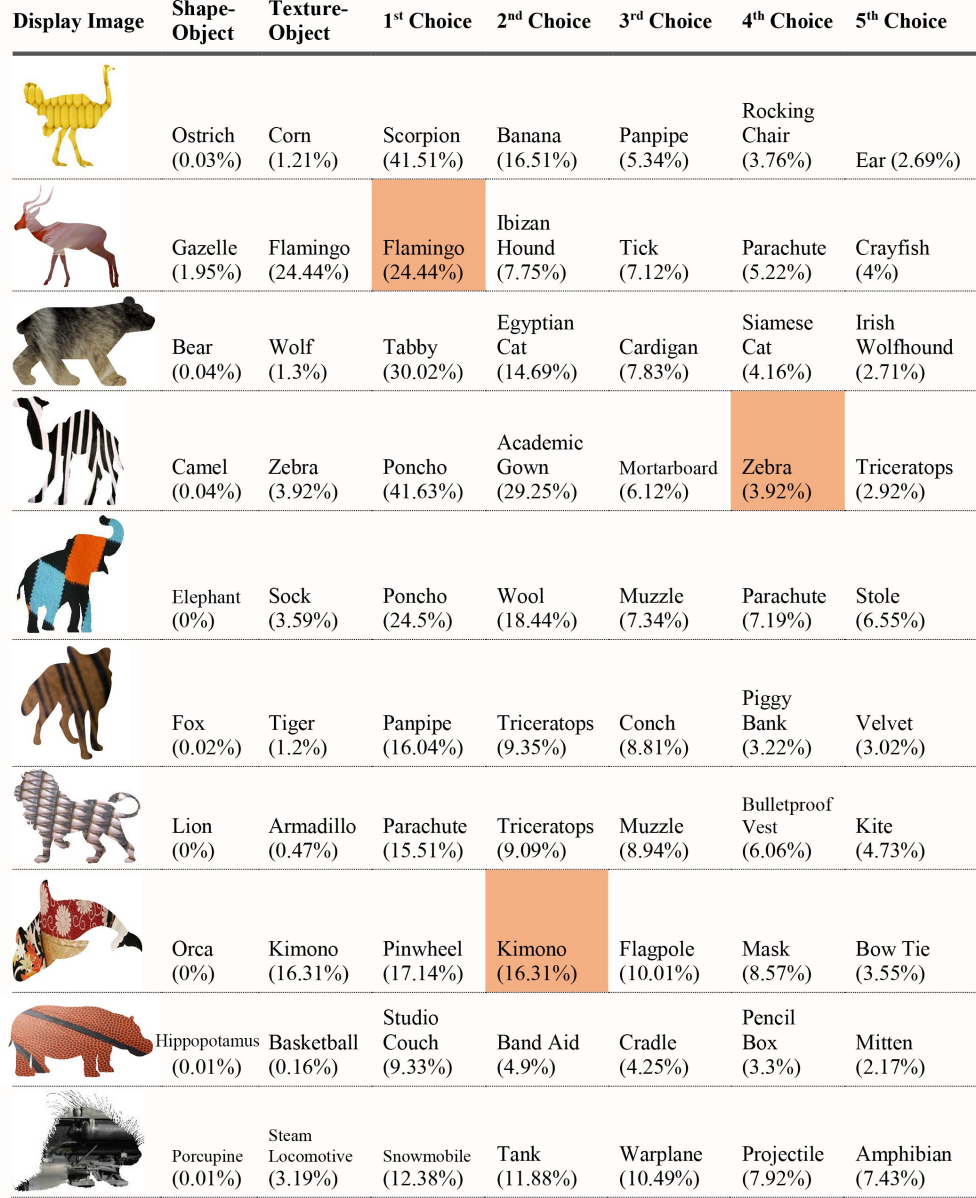
Network classifications for the stimuli presented in Experiment 1 Part 2.

**Fig 5:**
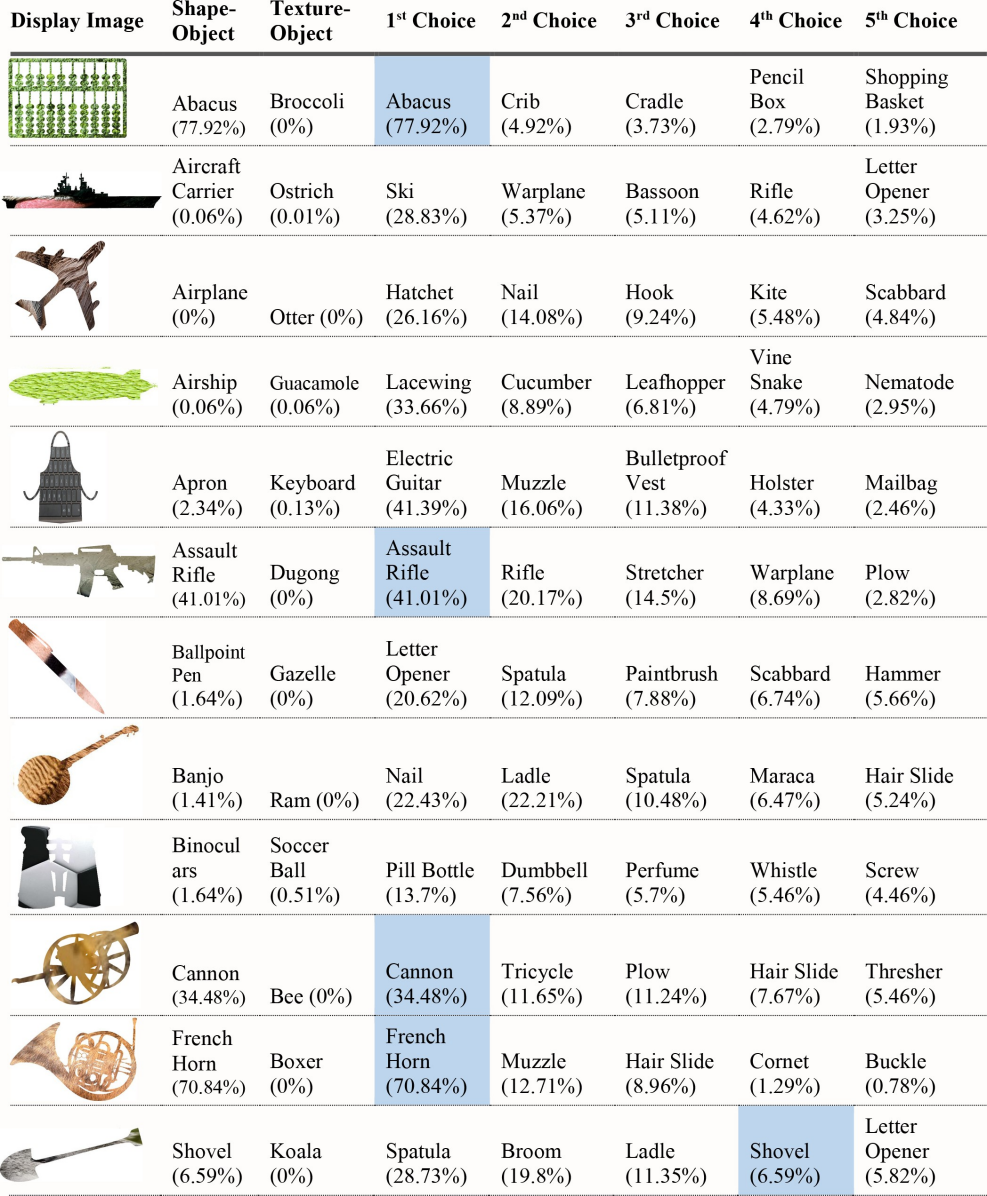
Network classifications for the stimuli presented in Experiment 1 Part 3.

**Fig 6:**
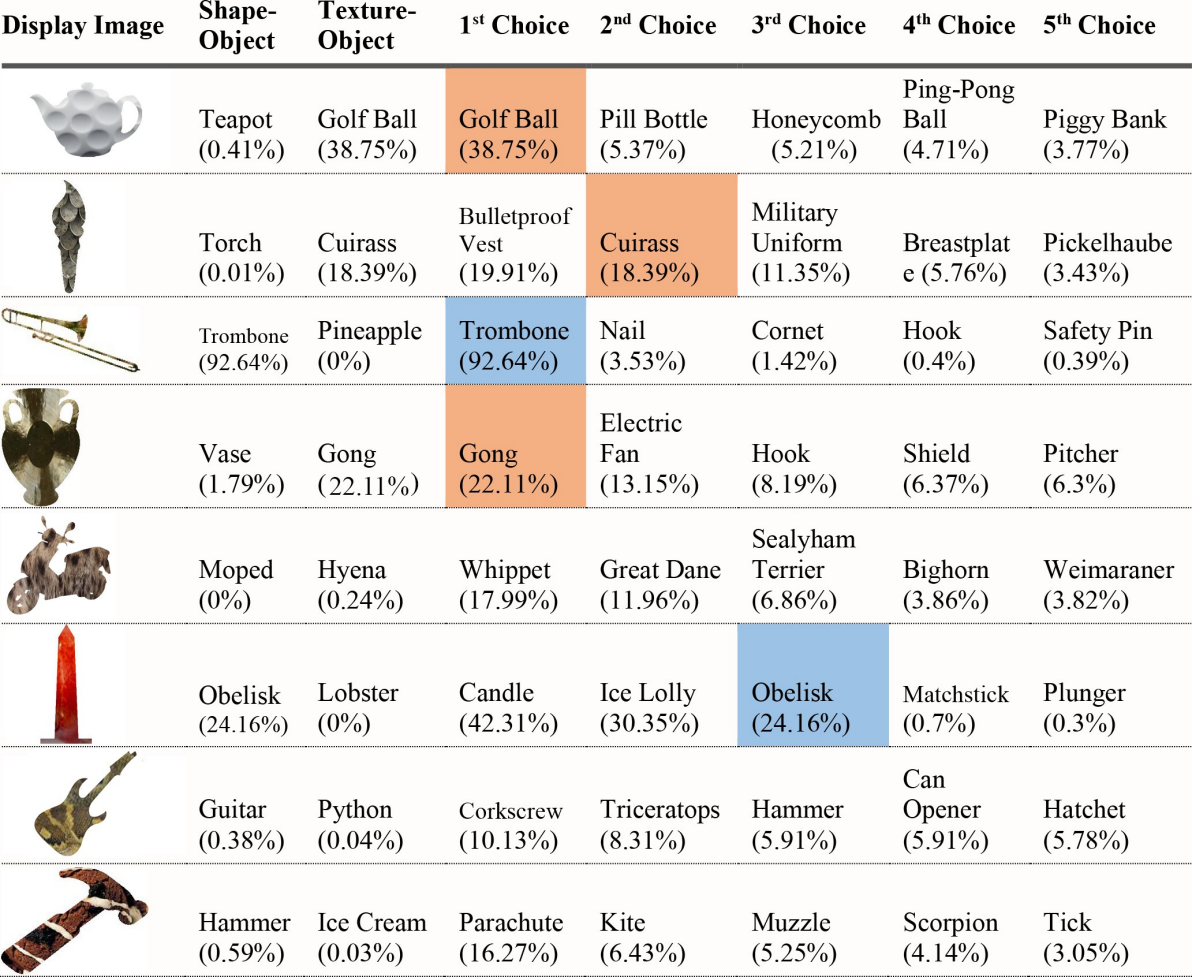
Network classifications for the stimuli presented in Experiment 1 Part 4.

For each of the displayed images, the network assigned a probability value to each of the 1000 object categories it had been trained to classify. The objects that received the five highest probability assignments for each image are shown, broken into four parts for size considerations, in Figs 3–6, along with the probability assigned to the correct shape and texture label. Based on shape, the network chose as its highest probability classification the correct answer for 5 of the 40 objects. Based on texture, the network chose as its highest probability classification the correct answer for 4 of the 40 objects. In terms of including the correct answer in its top 5 possibilities, the network classified 8 of 40 objects within its top 5 choices by shape and 7 of 40 objects within its top 5 choices by texture. Overall, the assigned probability was lower than is typical for natural images for both the correct texture-object label and the correct shape-object label. For photographs of objects that include texture, shape and context, 90% or more of the total probability across 1000 object categories will ordinarily be assigned to the correct object label. In this simulation, there were a few shape-based classifications that were near natural image performance, such as the abacus and the trombone, but on average shape-based classifications were nearer to 10%. Likewise, for textures, a few of objects were assigned probabilities that were 20% or greater but average performance was quite poor. Human observers, for whom shape is predominant in object recognition, readily produce correct shape labels for all of these objects, as confirmed by pilot studies. By contrast, across the whole display set used here, the object whose shape was depicted in the displays was selected by the network, on average, as its 209^th^ ranked choice.

Although there were indications of some use of shape information by the network for only about 20% of the displays tested in Experiment 1, an interesting pattern can be seen in the data. Use of shape information appeared to play some role in DCNN classification of artifacts but almost none for animals. The network had the object shape in its top-five classification selections for seven of the 20 artifacts, and only one of the 20 animals. The average probability assigned to the image’s shape label was 10 times higher for artifacts than for animals (17.90% vs. 1.75%). Texture appears to be about equally considered for both kinds of stimuli. There are three classifications in the top 5 choices for the texture-object in the 20 artifact images, and four in the 20 animal images; the mean probability assigned to the texture object is about equal for both kinds of images (3.22% vs. 3.73%).

**Fig 7:**
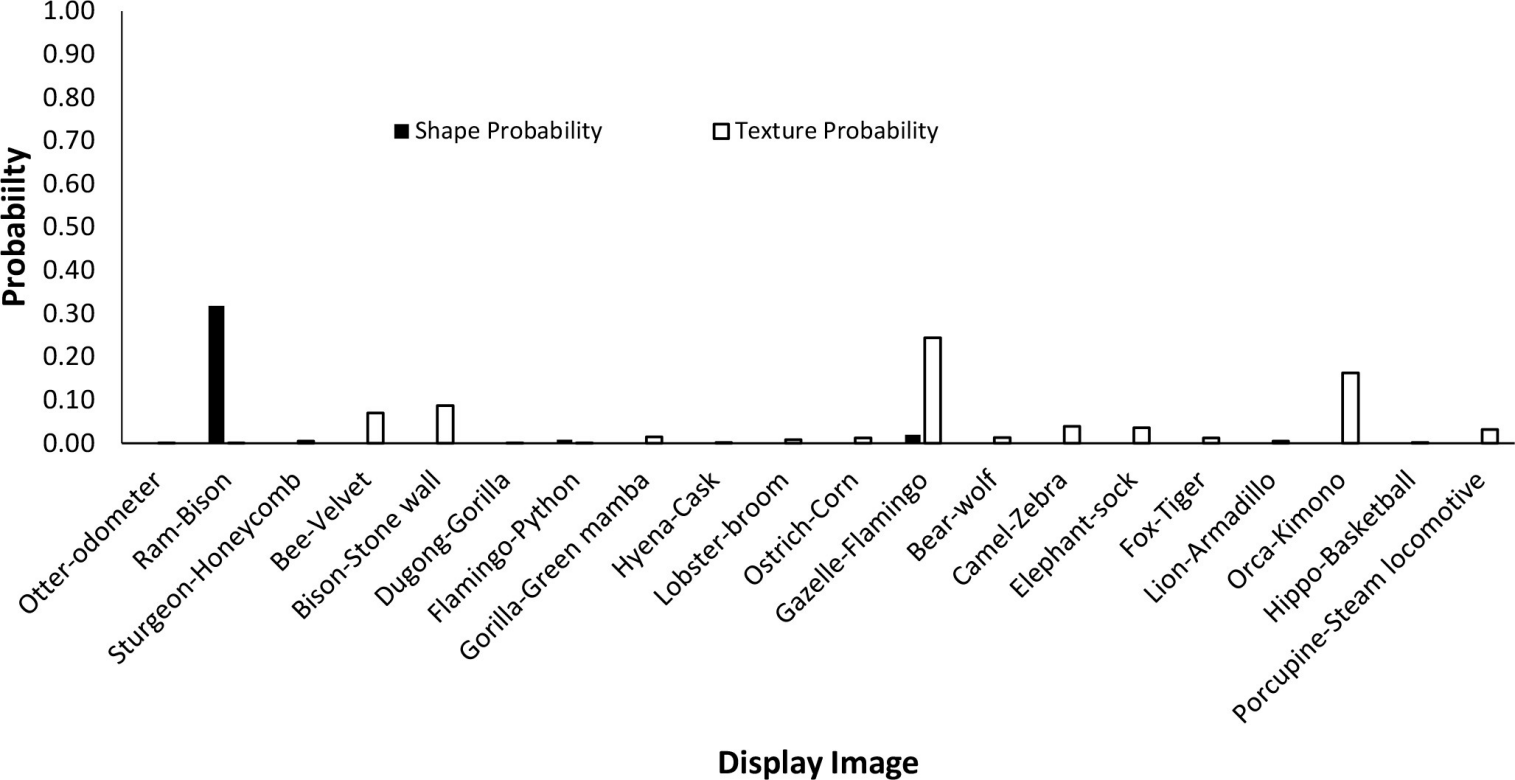
Comparison of probabilities assigned to image shapes and textures for animals. On the x-axis, the shape and texture of each object are given as shape-texture. Filled black bars display the probability given by the network to the correct shape. Outlined bars display the probability given by the network for the correct texture.

**Fig 8:**
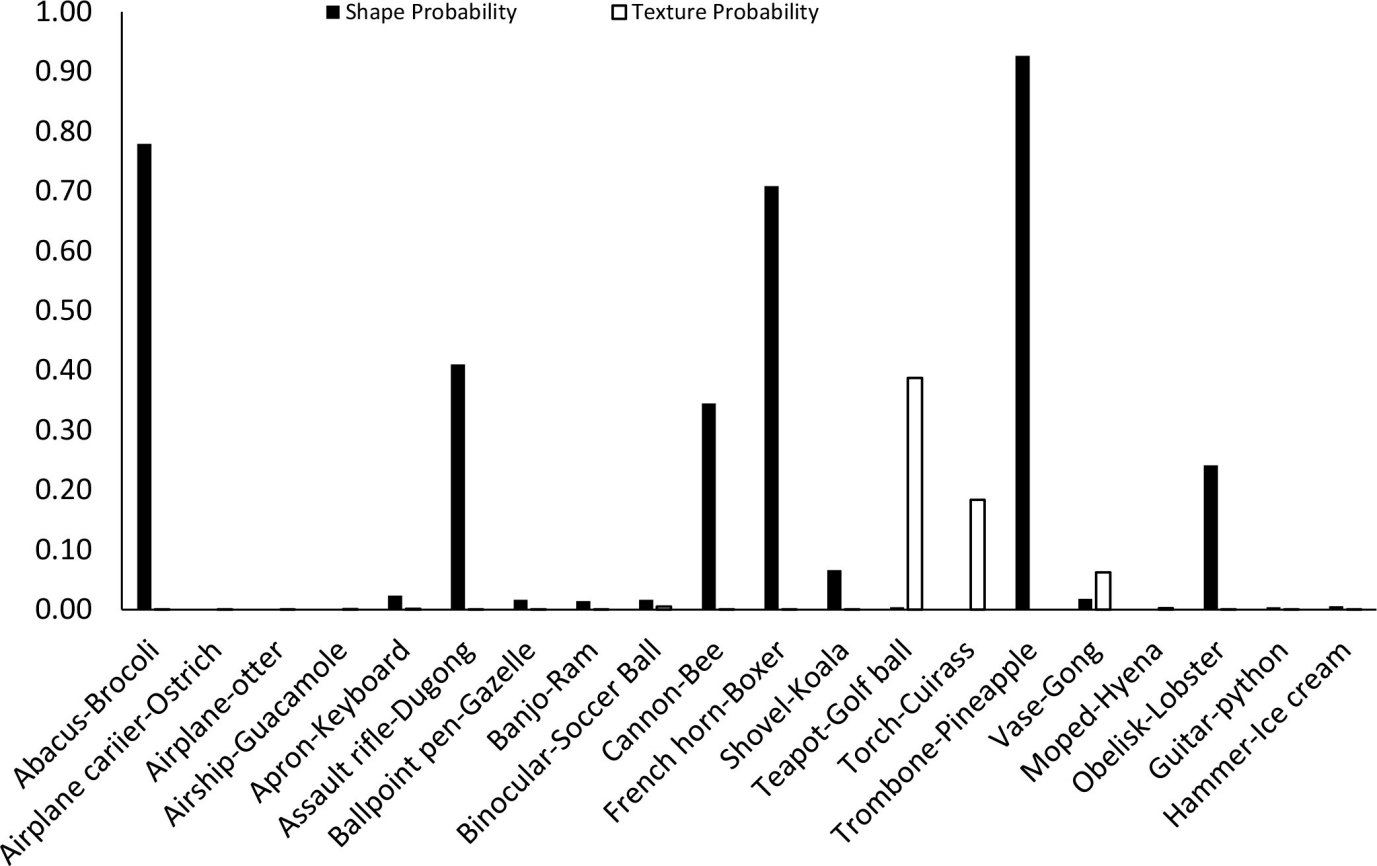
Comparison of probabilities assigned to image shapes and textures for artifacts. On the x-axis, the shape and texture of each object are given as shape-texture. Filled black bars display the probability given by the network to the correct shape. Outlined bars display the probability given by the network for the correct texture.

The data from Experiment 1 were also analyzed by directly comparing the probability value associated with the object whose shape was described by the silhouette and the object whose texture was overlaid atop the shape. Overall, texture was preferred more often than shape (23 vs. 17). However, there was a large difference between network behavior in manmade objects versus animals. The network assigned higher probability to the shape-label in 14 of the 20 manmade objects, but only three of the 20 animal images (see Figs 7 and 8).

Finally, we measured the contribution of shape and texture to network classification by looking at the rank order of the correct shape-object and the correct texture-object for each display used. For the correct shape response, the mean rank among network outputs was 86.70 for artifacts, and 330.50 for animals. For the correct texture response, the mean rank was 249.95 for artifacts and 65.30 for animals.

#### Experiment 1 Discussion

Experiment 1 suggested a major difference between human observers and DCNNs. Whereas human observers readily classify objects by shape, even in the face of uncharacteristic texture or context information, VGG-19 showed no evidence that shape information plays a primary role in DCNN classification. The correct label based on shape was chosen as the first choice classification by the network for only 5 of the 40 objects sampled, and the correct shape turned up on average as the 209^th^ ranked choice among network outputs in object classification.

Despite the lack of a clear, general accessibility of object shape in DCNN classifications, there was some evidence suggesting use of shape information in some cases. These cases were almost entirely confined to the 20 artifacts tested, in which 5 of 20 objects selected as first-choice classifications matched on shape, with 7 out of 20 objects placing the correct shape in the top 5 choices. In contrast, no first-choice classifications were correct for animal shapes, and only one of 20 animal displays showed any shape match among the top five classifications.

Although the network appears to utilize shape for classifying artifacts but not animals, there are still inconsistent examples in the test. Some objects, even from the artifact stimuli, do not show any evidence that shape is involved in network classification. For example, the airplane with otter texture was assigned essentially zero (.000002) probability to the airplane label (where .001 would be the value obtained by randomly guessing), instead classifying the image as “hatchet”, “nail”, or “hook” as its top three choices, none of which shares any shape similarity with an airplane. While this is a particularly glaring failure on the network’s part, over half of the artifacts were misclassified in all of the network’s top-five selections and the average rank order of the shape label of artifacts was 86.70. Many implausible shape misclassifications are assigned higher probability than the correct shape label. We mention this not to diminish the network’s success for some shapes on a task for which it was not explicitly trained, but to indicate that the results of Exp. 1 pose some differences in misclassification errors between the network and the human visual system. Humans have considerably less difficulty recognizing any of the objects by shape and would never consider some of the objects to which the network assigns high probability (e.g., “parachute” for hammer, or “electric guitar” for apron) as likely candidates.

Understanding why the network makes these kinds of misclassifications could be an important step to reveal the differences between network and human classification capabilities. It is possible that these erroneous responses are simply some intermediate landing point between shape and texture evidence, but classifications like “hatchet” for the airplane-otter suggest little consideration for the object’s texture in the network’s perceptual decision. It is also important to note that we tested objects separated from backgrounds and contexts. Typical tests of DCNNs include contextual information, which has been shown to be so important that networks perform reliably better than chance in classifying objects with images in which the object to be classified has been removed [21].

What could give rise to the differences we observed between artifacts and animals in networks that are not explicitly trained to recognize differences between superordinate categories like animacy? One possibility is that the network might learn to down-weight the contribution of texture cues in recognition of many artifact categories during training, since there is a more diverse range of textures and color associated with artifacts such as guitar and hammer. A sofa, for example, can be upholstered with any number of patterns, while a leopard’s fur will tend to be more consistent across exemplars. Another possibility is that DCNNs attribute less importance to shape cues in natural objects due to the large variability in the bounding contours of some natural objects. Animals are non-rigid, and their bounding contours vary considerably from image to image depending on pose, so it might be maladaptive to learn shape features for natural objects during training. Yet another possibility is that DCNNs really do not encode global shape but can nevertheless make use of some local shape features, which tend to be highly diagnostic for some artifacts, but provide little discriminability between different kinds of animals. Later experiments shed light on these possibilities. After considering additional results, we return to these issues in Experiment 4 and the General Discussion.

### Experiment 2

Experiment 1 showed that in displays that preserved overall object shape but altered their texture, shape was a poor predictor of network classifications. There was some indication, however, of sensitivity to shape information in certain cases. The network made several accurate classifications of objects with non-canonical surface texture. This success appeared to be largely confined to artifacts, although even artifact classifications included many implausible top selections. On the other hand, shape information appeared to be largely irrelevant for classification responses generated for animal displays. In Experiments 2–4, we developed more detailed tests to examine whether networks could classify objects only based on shape with changed or absent surface texture and context information.

It is a remarkable fact, one attesting to the primacy of shape processing in human perception, that human observers readily recognize shapes in arbitrary materials (and construct and display them, etc.). In Experiment 2, we presented two deep networks with glass figurines. All figurines were pictures of real glass objects. Since glass figurines lack the natural surface colors and textures of the objects represented, we expected that accurate classification would be difficult without a representation of the object’s bounding shape. We expected that if the networks did have access to object shape, they might be able to accurately classify the glass figurines even in the absence of other, usually accompanying, cues for recognition. In other words, DCNN classifications that would resemble even a child’s intuitive response the first time they see a glass elephant would furnish evidence that shape information plays a role in DCNN classification.

#### Experiment 2 Method

**Fig 9:**
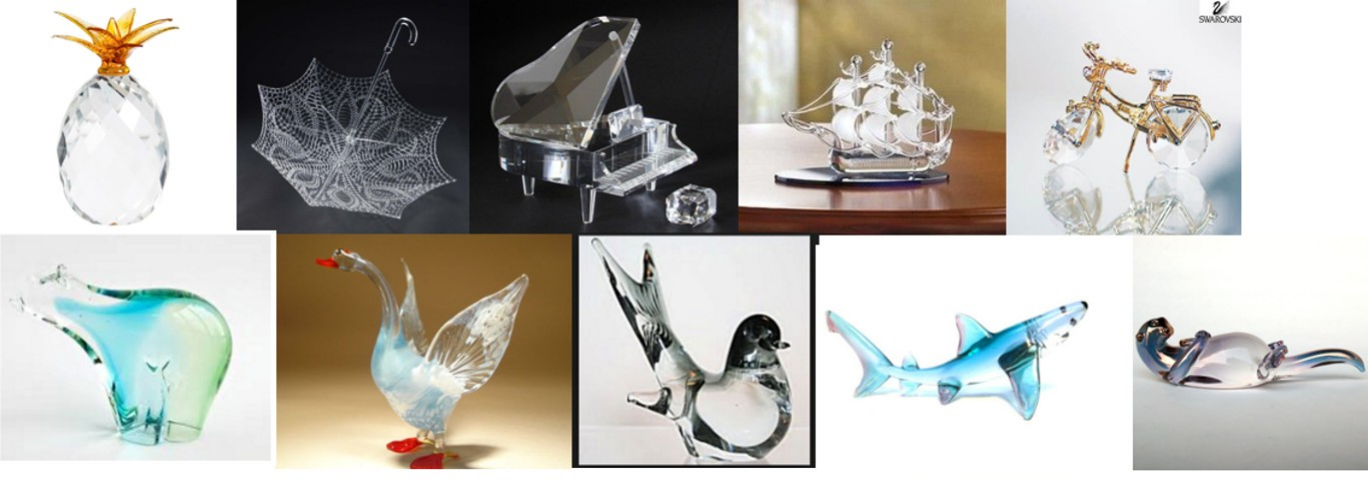
Sample stimuli used in Experiment 2.

*Images*. Twenty images of glass figurines were found from the internet (see S2 File for URLs). Half of the figurines were of animals and half were of manmade objects. The images had some texture information, but the overall texture of each object was very different from a canonical instance of the represented object. The background in all but one of the 20 images was non-descriptive; either a homogeneous field or a color gradient. The one exception is the schooner figurine (see below), which is photographed on a table. See Fig 9 for examples.

*Network*. classification was tested on two networks: AlexNet, with seven layers, and VGG-19.

#### Experiment 2 results

**Fig 10:**
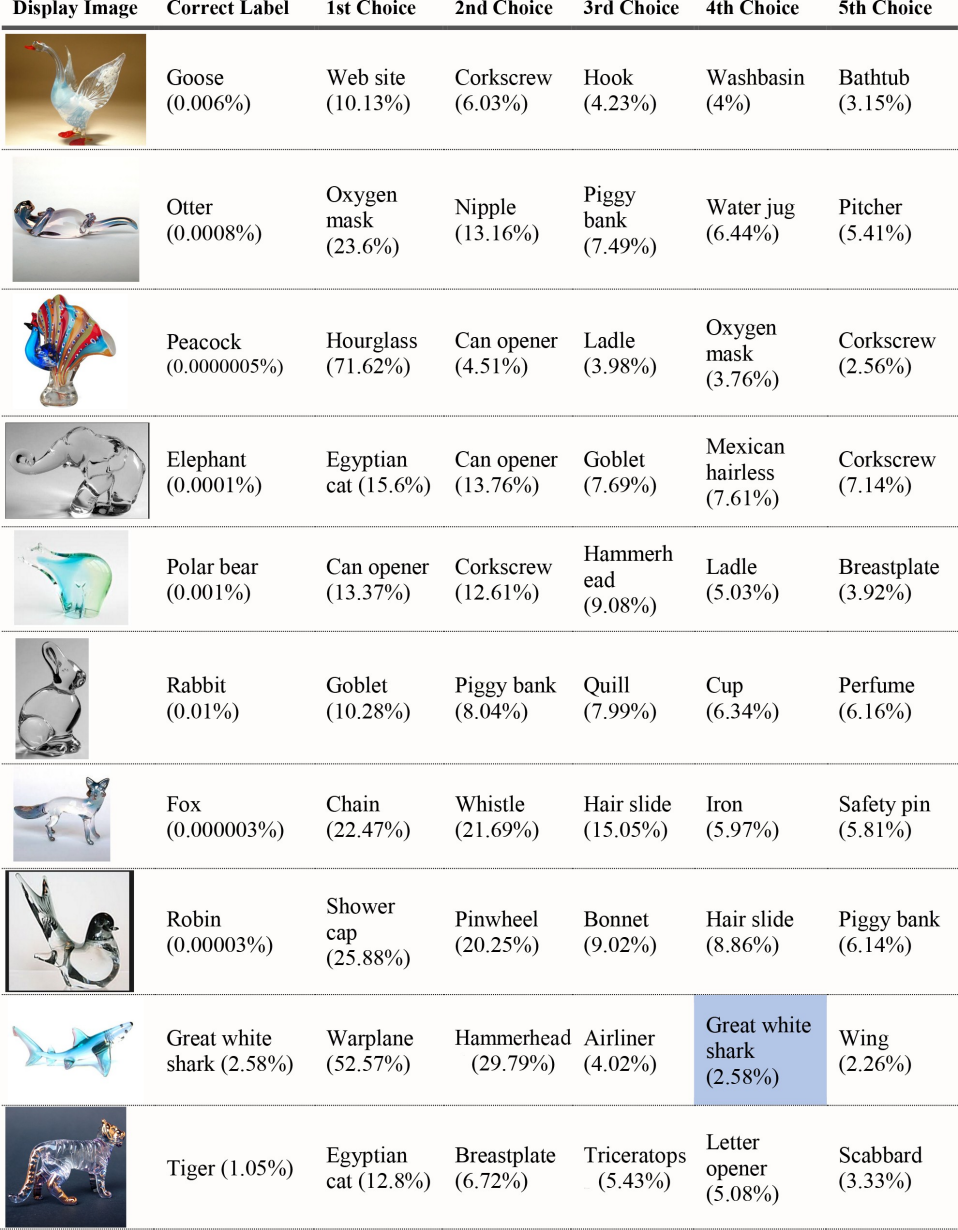
VGG-19 classifications for glass figurines Part 1. The leftmost column shows the image presented to the VGG-19 DCNN. The second column shows the correct object label and the probability generated by the network for that label. The other five columns show probabilities for the network’s top five classifications, ordered left to right from highest to lowest. Correct classifications are shaded in blue.

**Fig 11:**
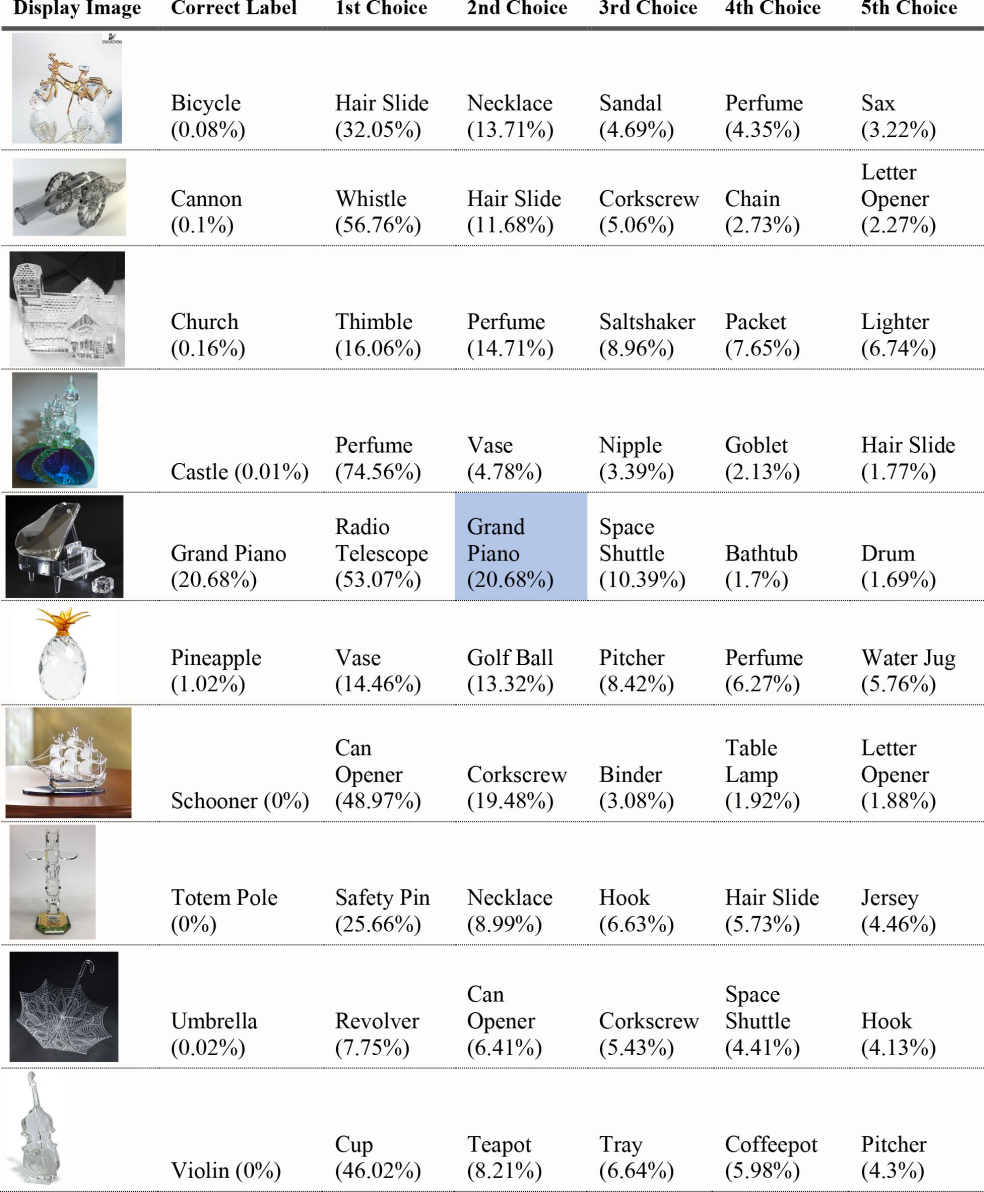
VGG-19 classifications for glass figurines Part 2.

We assessed the networks as correct if they generated the names that a human observer would give to each image. (Human classification was verified in pilot work with human observers.) Neither network produced as its top choice the correct label for any of the 20 objects. Figs 10–11 show the top five classification choices for the 20 images shown for VGG-19 (the better-performing network; see below). Percentages in parentheses represent the probability assigned to each label by the network. In the absence of any evidence, the baseline probability of an object was 0.1%, as there were 1000 object categories.

Most of the top-choice responses seem bizarre for human perceivers, such as "web site" for goose, "oxygen mask" for otter, "can opener" for polar bear, and "chain" for fox. Although the stimuli here are (intentionally) different from what the networks were trained on (because they are glass figurines), the results clearly indicate that shape, if accessible at all by DCNNs, does not play the defining role in object recognition that it does for human perceivers. Comparing the networks' responses to chance level performance (0.1%), AlexNet assigned a probability below chance to the correct shape for 18 of the 20 test objects, and VGG-19 assigned a probability below chance to the correct shape for 15 of the 20 objects. Analysis of the rank order of correct labels revealed that the correct shape choice averaged, across the display set, a mean rank of 162.60.

The criterion of finding the correct answer among the top five most probable responses is often used to assess performance of DCNNs. Using this criterion, VGG-19 correctly classified two of the 20 images (the shark figurine and the grand piano figurine), while AlexNet misclassified all 20 images. VGG-19 assigned a 20.58% probability to the correct grand piano response, second only to its 53.07% probability assigned to "radio telescope." For the shark, VGG-19 selected the correct label as its 4th choice, assigning a 2.58% probability. For many of the remaining images, the objects were misidentified as glass-made or metal-made kitchen objects, such as “water jug”, or “can-opener”.

As another way of measuring the network’s sensitivity to object shape, we compared the probability the network gave to the object in the image with the probability it gave to the nine other objects that were used in the experiment. For example, we compared the probability that the network gave to “goose” when it was shown a goose figurine to the average of the probabilities the network gave to the other nine animal labels (“otter”, “peacock”, and so on) for the same figurine. For this analysis, we kept glass animals and glass objects separate to ensure that higher probabilities could not be accounted for by low-level contour features like the presence or absence of a straight edge. For both animate and inanimate objects, probabilities were not higher for the correct shape-label than for the average of the other nine incorrect shape labels more often than would be expected by chance. Five of the 10 inanimate objects, and seven of the 10 animate objects were given a lower probability than the average of the other nine in their class.

#### Experiment 2 Discussion

The networks showed little capability of classifying glass figurines. Although glass figurines of animals remove the natural surface texture, they also introduce surface properties of their own. As in Experiment 1, surface features appear to play a major role in the network’s classification decisions. For example, the peacock figurine has “water jug” and “pitcher” in its top-five objects, despite having no shape similarity to either. “Goblet”, “vase”, “cup”, and “hourglass” are also common misclassifications made by both AlexNet and VGG-19. By contrast, the networks appear to make few misclassifications based on similarity between the shape of two objects. Aside from the piano and great white shark, only the tiger had a misclassification (“Egyptian cat”) that would be consistent with use of some sort of information about object shape. It appears that shape does not play an independent, predominant role in recognition, as it does in humans [28–30].

Do the network’s few successes point to a broader trend that the network is utilizing both shape and texture in its classification decisions? It is difficult to determine what is different about these three images than the other 20 images that the network fails to classify. One thing to keep in mind is that there is a chance that a plausible shape classification appears in the network’s top-five selections without the network having any sensitivity to the shape of the image. The tiger seems like a likely candidate for this possibility. While Egyptian cats and tigers have some shape features in common, Egyptian cats and elephants have very few, but the network names “Egyptian cat” as its top selection for both the elephant and the tiger glass figurines. In fact, it assigns higher probability that the elephant is an Egyptian cat than that the tiger is an Egyptian cat. It is possible that some surface feature, or conjunction of surface features and local edge properties, is driving classification in both cases.

**Fig 12:**
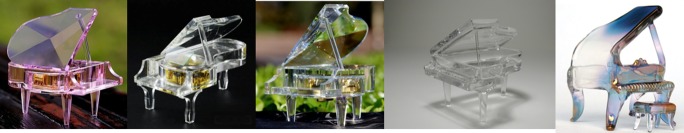
Five additional glass Pianos. VGG-19 incorrectly classified each of these five images despite correctly classifying the glass piano shown in Fig 11.

This explanation is less satisfactory for the grand piano and great white shark, whose shape label is actually in the top-five selections and does not appear in any of the other images’ top selections. In the case of the piano, one possibility we considered is that the texture of the keys drove classification, but a further test showed that the network performs well even after the keys have been occluded or blurred out. We tested the network on five additional glass grand piano images (see Fig 12), and it was unable to correctly classify any of them in its top-five selections. It is unclear what information is present in the image where the network does well that is absent in the other five, but it is likely a local shape feature, as global shape is very similar across the six images. Likewise, it is likely that local contour features, not global shape, are driving the network’s accurate classification of the great white shark. We discuss this hypothesis in greater detail and revisit these positive examples in Experiment 4, after we have considered more data regarding the network’s sensitivity to local and global shape information.

The results of Experiment 2 clearly showed an absence of shape sensitivity for glass figurines. Human classification of such objects is affirmed by the fact that we make, display, and recognize such objects routinely. Although in natural scenes, an elephant is never made of glass, is never 4" high, and only rarely appears on anyone's desk or coffee table, human use of object shape makes recognition of a glass elephant on a desk effortless and routine. Not only is this predominance of shape not seen in DCNN performance, there is little to suggest that shape, independent of other information, is accessible at all in classification of these objects.

In this experiment, texture or surface quality information provided a stronger influence than object shape on object recognition by both AlexNet and VGG-19. We might suspect that the strength of surface texture cues pulled the networks’ top-five classifications towards texture-object labels. If object shape played any role at all, however, we would expect that the correct object label would be assigned a probability that is at least greater than chance. In most cases, the correct shape label was assigned a value less than chance, and objects of similar composition but different shapes tended to receive probabilities as high as the correct shape.

### Experiment 3

A remarkable fact about human object perception is that we readily extract shape from outline drawings. This ability clearly depends on shape, as outlines omit surface information completely. Object outlines have the same texture within the bounding contour as outside it, and there is no variation in texture between the outlines of two different objects. We tested outlines in Experiment 3 to extend the earlier results and specifically to remove competing texture or surface information as much as possible. If, for example, the pictures of glass figurines somehow distracted deep networks from utilizing some encoded shape information due to competing surface texture, we expected that the problem would be substantially mitigated by using outlines. On the other hand, if deep networks cannot access shape from outline drawings of objects, we expected poor performance.

#### Experiment 2 Method

**Fig 13:**
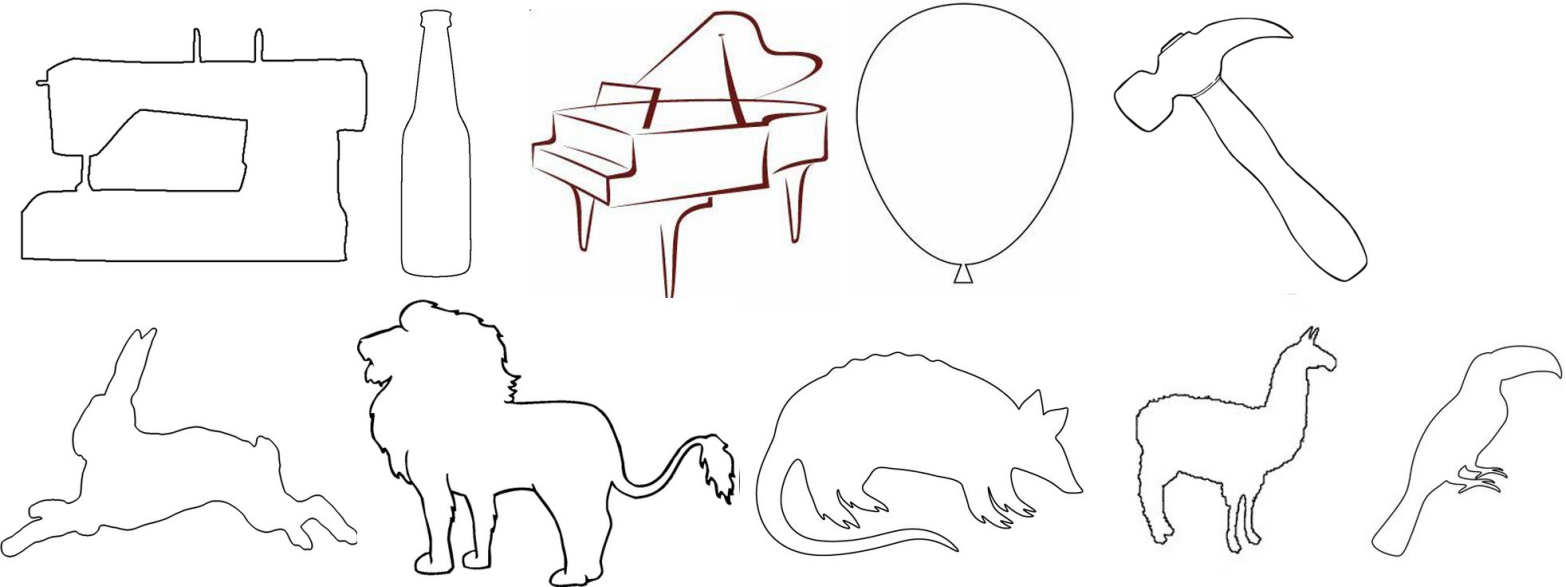
Sample outline stimuli used in Experiment 3.

*Images*. Forty images of object outlines were selected from the internet, half of which were manmade artifacts, and half of which were animals. In all images, the only contrast difference was at the object contour. Images were uniformly white at all other locations in the image. There was a degree of abstraction in the object contours, as the outlines were not boundaries of real natural objects. All were readily recognized and correctly named by human observers. Fig 13 shows examples of stimuli used in Experiment 3.

**Networks:** Tests were conducted using both AlexNet and VGG-19.

#### Experiment 2 results

**Fig 14:**
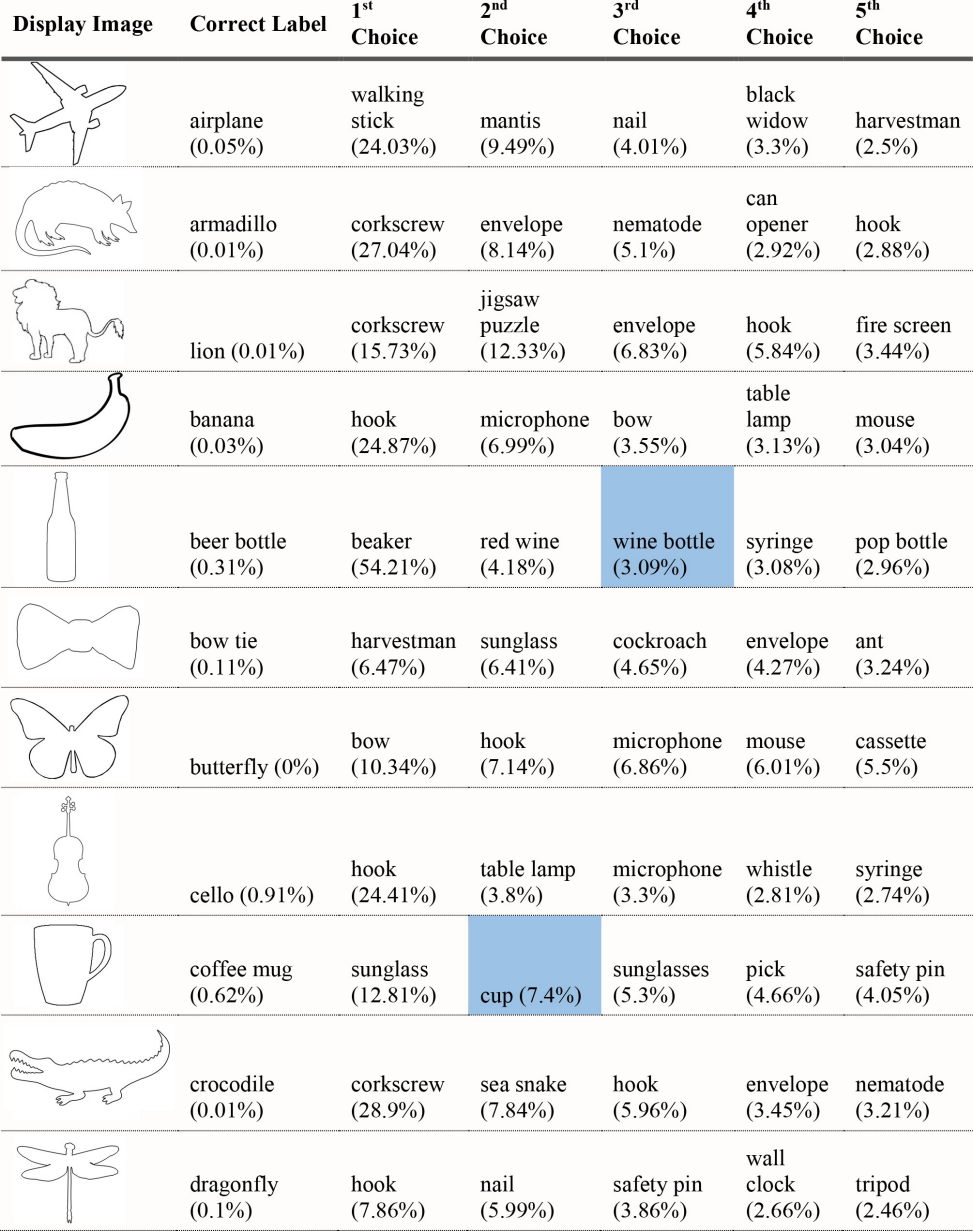
VGG-19 classifications for object outlines Part 1. The leftmost column is the image presented to the DCNN. The second column from the left is the correct object label and the classification probability produced for that label. The other five columns show probabilities for the VGG-19’s top five classifications, ordered left to right in terms of the probability given by the network. Correct classifications are shaded in blue.

**Fig 15:**
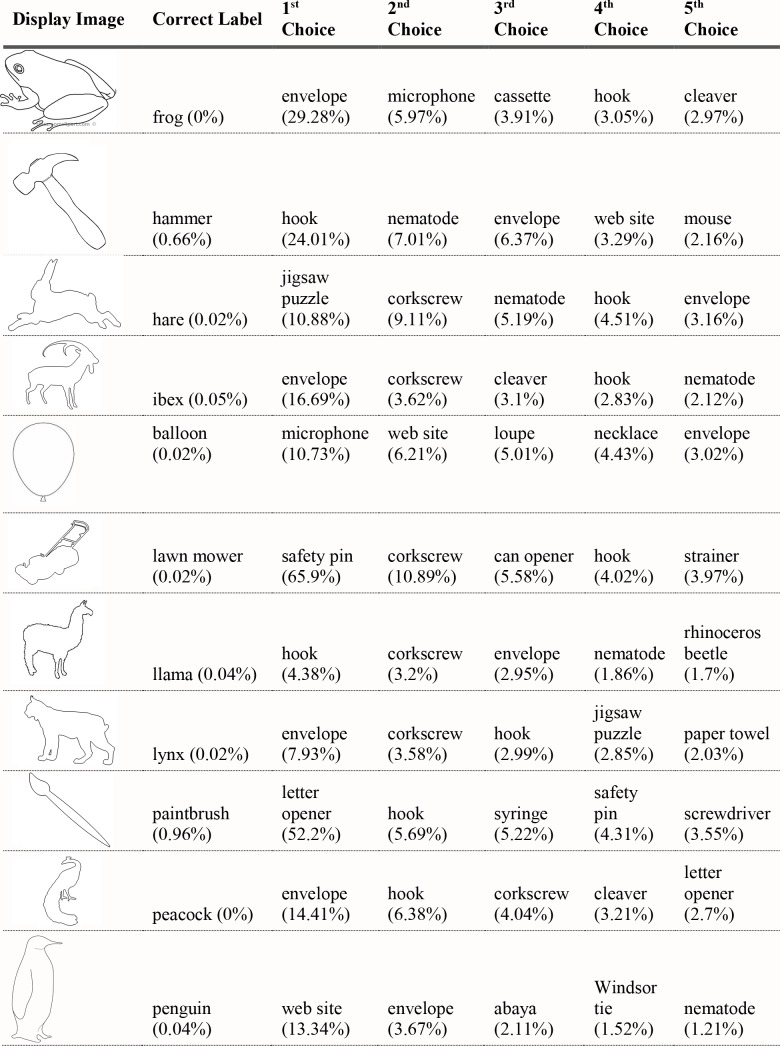
VGG-19 classifications for object outlines Part 2.

**Fig 16:**
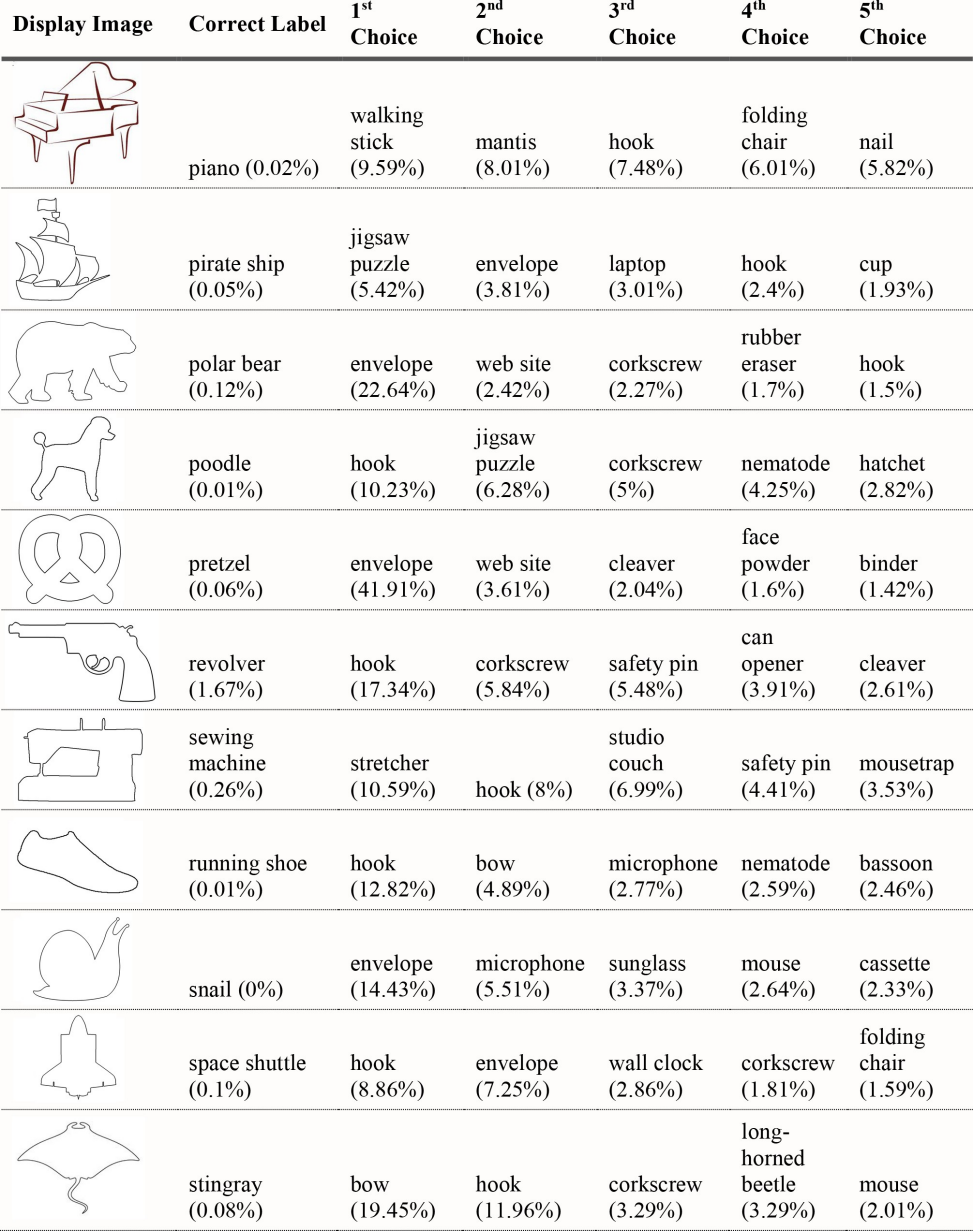
VGG-19 classifications for object outlines Part 3.

**Fig 17:**
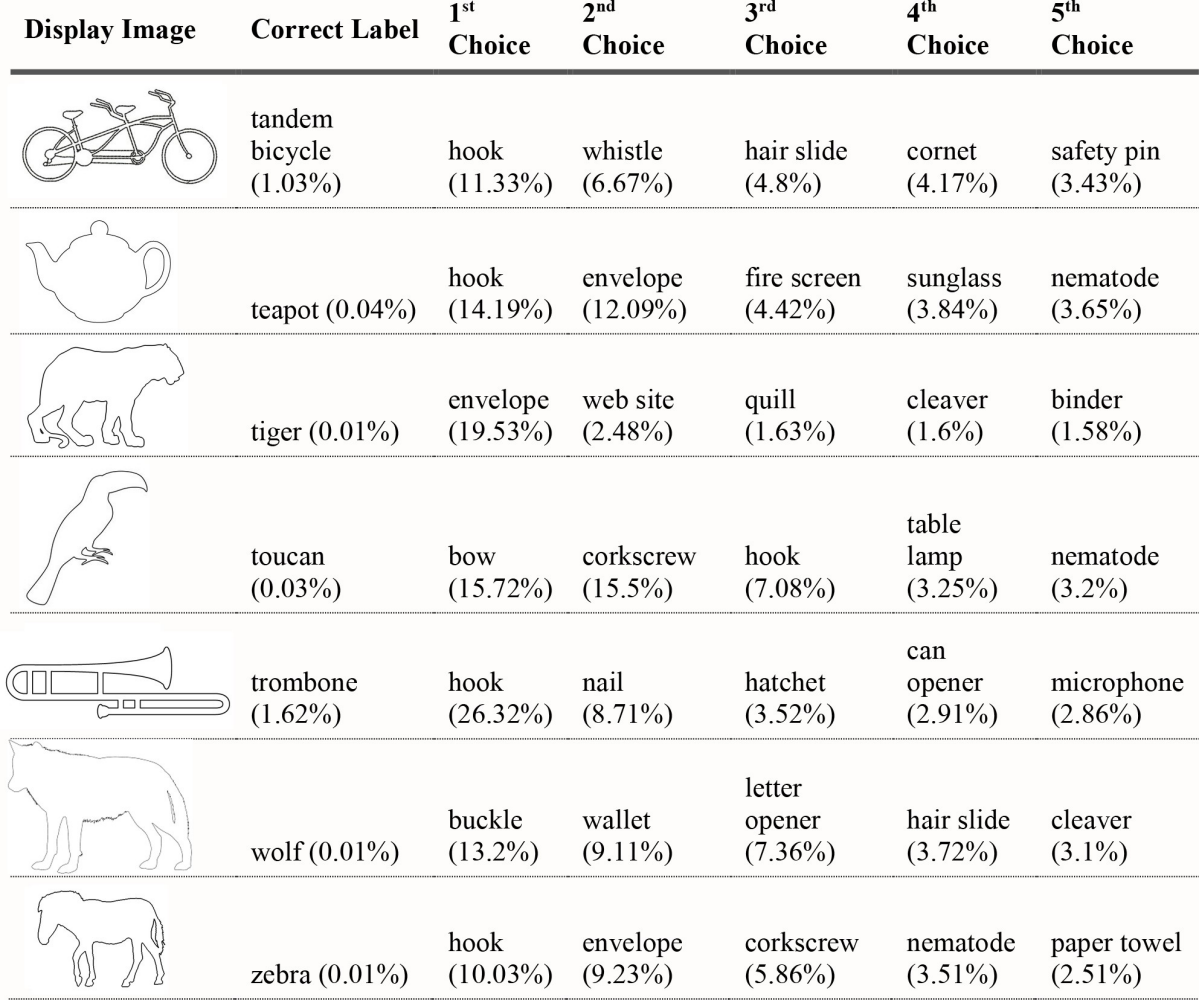
VGG-19 classifications for object outlines Part 4.

None of the correct shape labels for the 40 objects were chosen as the first-choice classification by either VGG-19 or AlexNet. Two of the 40 objects were named among the top-five choices for VGG-19, and only one was given as a top-five response by AlexNet. Figs 14–17 show the full set of responses. Twenty-eight of the probabilities assigned to the object label given by humans were below the chance rate of 0.1% in VGG-19, and 35 out of 40 were below 0.1% in AlexNet. On average, the correct classification by shape was the network’s 328^th^ most preferred choice. As in Experiment 2, we compared the probability assigned to the correct shape label to the shape label of 19 other animate or inanimate objects. For animals, the probability was lower for the shape-object than for the mean of 19 other animal images in eight of the 20 trials. For artifacts, it was lower in 10 of the 20 images. These results do not differ from the chance likelihood that the target shape will be higher than the mean of the other 19 in half the trials. Of the objects that were correctly classified, all were artifacts and none were animals.

For some reason, "corkscrew" was the VGG-19's first or second choice for 10 of the 40 objects, and it appeared among the top 5 choices for 15 objects. More understandable, perhaps, is the finding that "envelope" was the first or second choice for 14 of the objects, and this response appeared in the top 5 for 19 objects. These responses are most likely due to the local similarities between training photographs of a white envelope, possibly including black letters, and the tested outline images, which are also white with thin black lines. There is no evidence that the shape of the outline images is being extracted and classified using shape features acquired from training images with similar figure boundaries.

#### Experiment 2 Discussion

Deep convolutional networks showed little capability to recognize objects with shapes defined by outline contours. Whereas the glass figurines had textures that were compatible with a different set of objects from their correct shape-object label, the texture in object outlines is not differentially diagnostic of any object classification, as it is identical to the surrounding background and identical throughout the object set. We hypothesized that if global shape information is extracted by the neural networks but was not used for glass figurines due to the strength of texture cues, object shape might play a larger role for outline images. The data do not support this hypothesis; classification performance was just as poor for object outlines as it was for pictures of glass objects.

The failure to classify objects correctly based on outline shape marks a clear divergence from human perception. All of the displays used in this experiment were consistently and accurately classified by human observers. We verified this with behavioral tests in which the outlines were shown together, as well as interleaved with photographs to confirm that humans have no trouble classifying object outlines, even in unexpected contexts. However, the results do not by themselves exclude some possibilities for use of shape information in DCNNs. That humans readily see shape in outline drawings is a remarkable fact in itself, and one that is not completely understood [31, 32]. It would certainly be possible to envision a shape processing system that balked at outlines and used only surface edges. As introductory drawing teachers often stress, outlines are ecologically anomalous. Within biological vision, some perceptual processes seem to treat outlines and surface edges differently, as in perceptual completion [33]. Humans can see shape given by ordinary surface edges or outlines, but if deep networks cannot utilize the latter, it does not necessarily imply that surfaces edges do not play some role in classification in conjunction with texture features. Humans' fluent use of outlines to see object shape indicates the strong role of shape representation in human object recognition, and human interpretation of forms in outlines probably connects naturally to some stage of perceptual representation [34]. It appears that DCNNs differ from human processors in that they have little or no linkage between shape properties embodied in outlines and the classification labels in the output layer. DCNNs’ failure to use outlines does not, however, rule out the possibility that these systems may utilize some shape information in more natural cases.

These issues may relate to an important factor not yet mentioned. Figure-ground assignment, or equivalently, assignment of border ownership at occluding edges, is a well-known feature of human perceptual organization [35, 36]. It appears that outlines, especially closed outlines, are interpreted in human vision as owning their borders. (The enclosed area is taken to be the bounded object.) DCNNs do not have an obvious way of representing figure vs. ground or border ownership. These seem to be more explicitly representational aspects of human perceptual processing. At least some of the problem with outlines may involve figure-ground issues. Misclassifications of objects as “hook”, “safety pin”, and “syringe”, which all have empty interior regions, suggest that DCNN's might be interpreting the actual outline as the object, rather than seeing an object as occupying the region within the outline.

On the other hand, results for a few of the images presented in Exp. 3, the beer bottle and the coffee mug, as well as a few objects like the tandem bicycle and trombone which are not correctly classified but are assigned probabilities significantly greater than chance, suggest that deep networks do not exclusively treat the black outlines as bodies of the objects themselves. If the networks were only treating the black outlines as the figure, these objects should have below-chance probability, as they do not have thin, black forms. Of course, we do not mean to imply that a DCNN employs any consistent approach or strategy; any good predictor from any of the many filters in the network, may influence the outcomes toward a correct classification. Perhaps these images have certain local features that can be extracted and further facilitate the recognition. DCNNs may not capture global shape, but may pick up some relatively local shape features, a possibility we discuss further in connection with later results.

### Experiment 4

Experiments 2 and 3 found little evidence that deep convolutional networks access global shape in object recognition tasks. These results may seem surprising, as some recent reports have suggested that DCNNs do possess some shape classification abilities. Kubilius et al. [18] found that removing surface features from the Snodgrass and Vanderwart dataset of colored-in line drawings [37] did not totally destroy networks’ classification performance. With the regular line-drawing images, classification performance was 80–90%. Removal of color information reduced performance to around 70%, and removal of all inner surface gradients (black silhouettes) brought performance down to about 40%. It is arguable whether 40% classification accuracy represents a success or failure in shape-based classification for DCNNs. On the one hand, this marks a divergence from human performance, which is largely unaffected by the removal of color and inner surface gradient information from most objects. On the other hand, it seems almost impossible that the network would reach even 40% accuracy without some information about object shape. In contrast, our findings about recognition for glass objects and line drawings provided almost no evidence that shape representations are used for classification in DCNNs. In Experiment 4, we tried to replicate Kubilius et al.’s findings as a first step in clarifying this apparent discrepancy.

#### Experiment 3 Method

**Fig 18:**
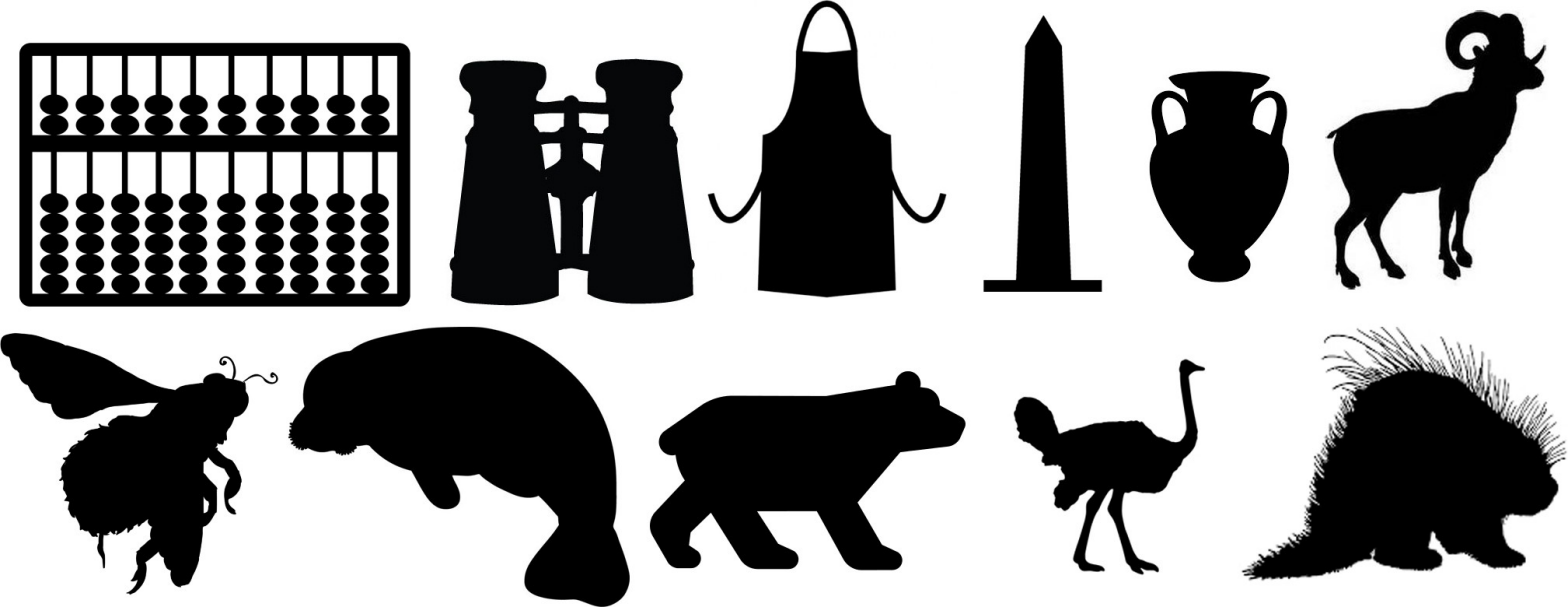
Sample stimuli used in Experiment 4.

*Images*. The same 40 object silhouettes found on the internet and used in Experiment 1 were used in Experiment 4, this time without any texture substitution. All images consisted of a single black figure on a white background. Half of the images were artifacts, and half were animals. The black figures were silhouettes of object drawings, rather than being taken from photographs of real objects with their textures removed, so some contour information that would typically be in a natural instance of the object was abstracted in the silhouette images. These abstractions have no effect on the objects’ recognizability to a human observer. See Fig 18 for examples. We also tested the network on the same 40 silhouettes with white figures on a black background and red figures on a white background to measure the influence of surface color on network classification performance.

*Networks*. As in Experiments 2 and 3, we tested AlexNet and VGG-19 on the silhouette images.

#### Experiment 3 results

**Fig 19:**
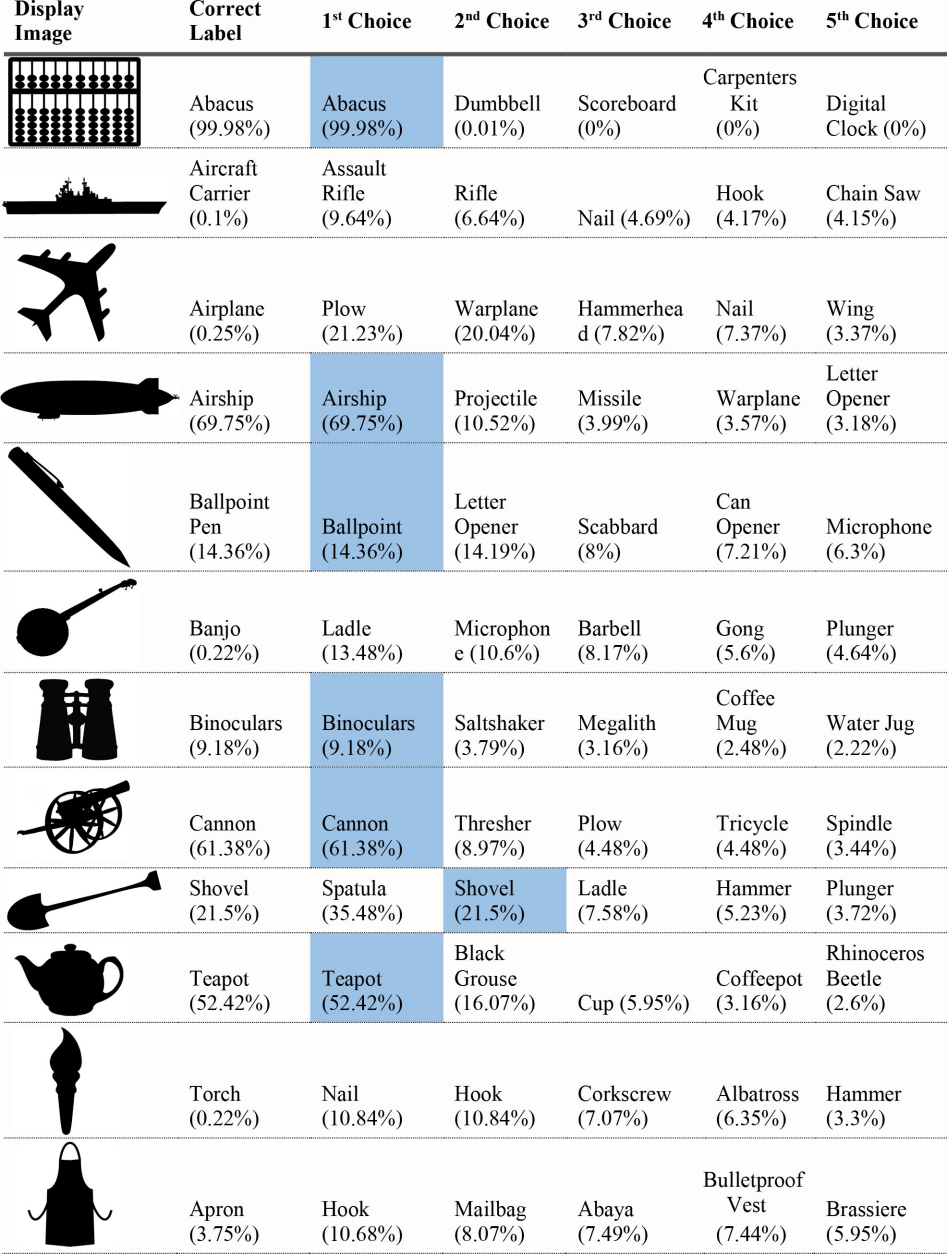
VGG-19 classifications for black object silhouettes Part 1. The leftmost column shows the image presented to VGG-19. The second column from the left shows the correct object label and the classification probability produced for that label. The other five columns show probabilities for the network’s top five classifications, ordered left to right in terms of the probability given by the network. Correct classifications are shaded in blue.

**Fig 20:**
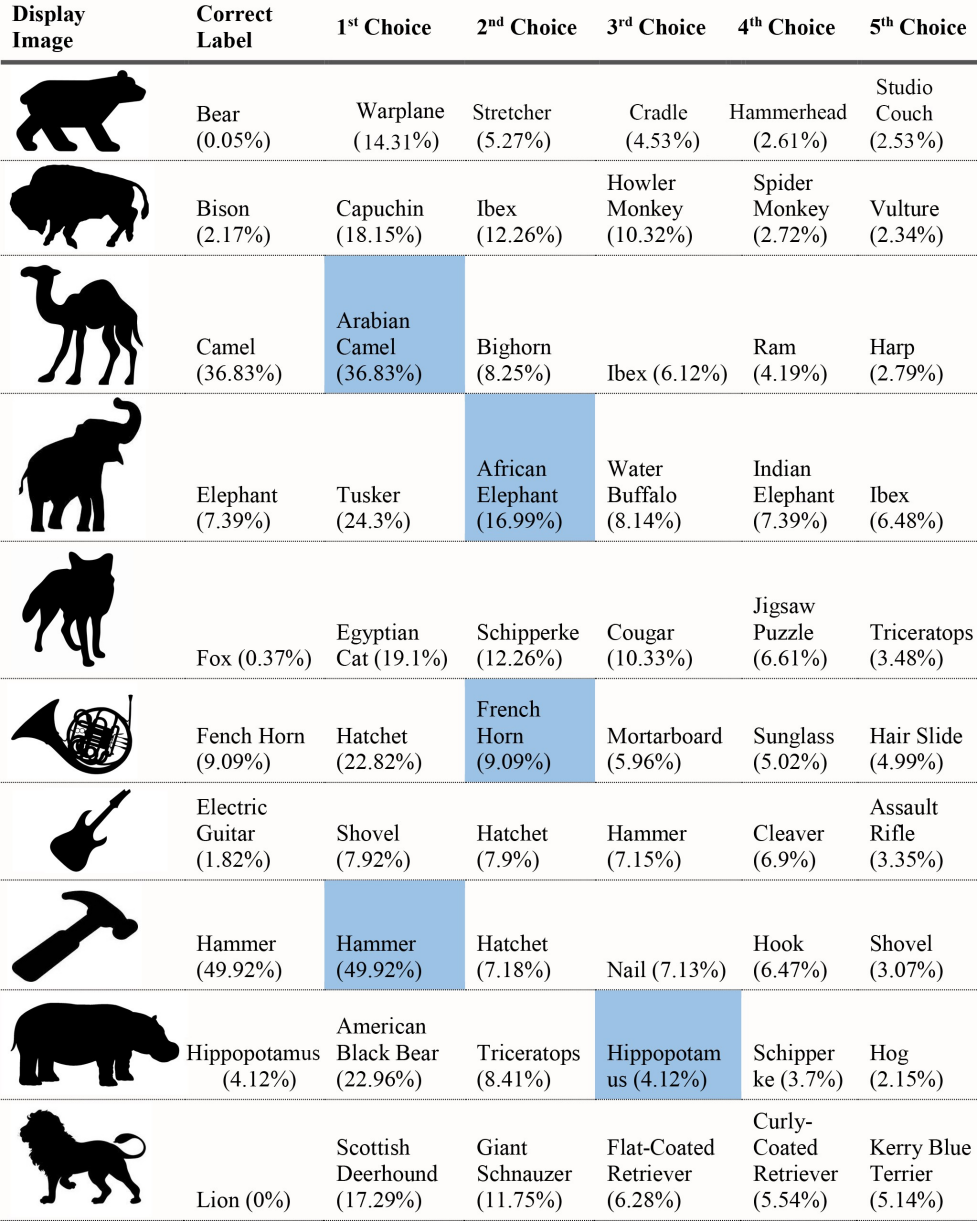
VGG-19 classifications for black object silhouettes Part 2.

**Fig 21:**
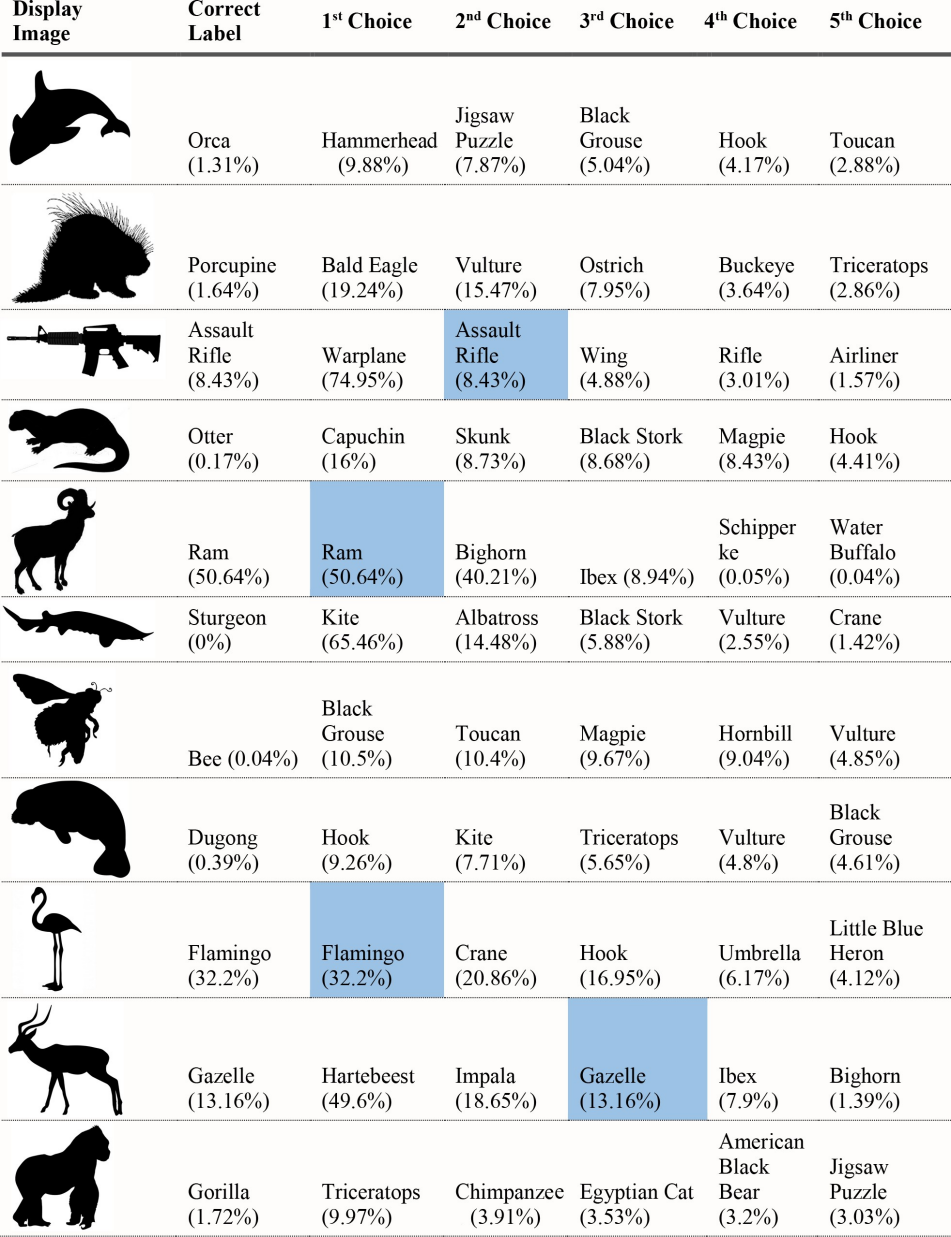
VGG-19 classifications for black object silhouettes Part 3.

**Fig 22:**
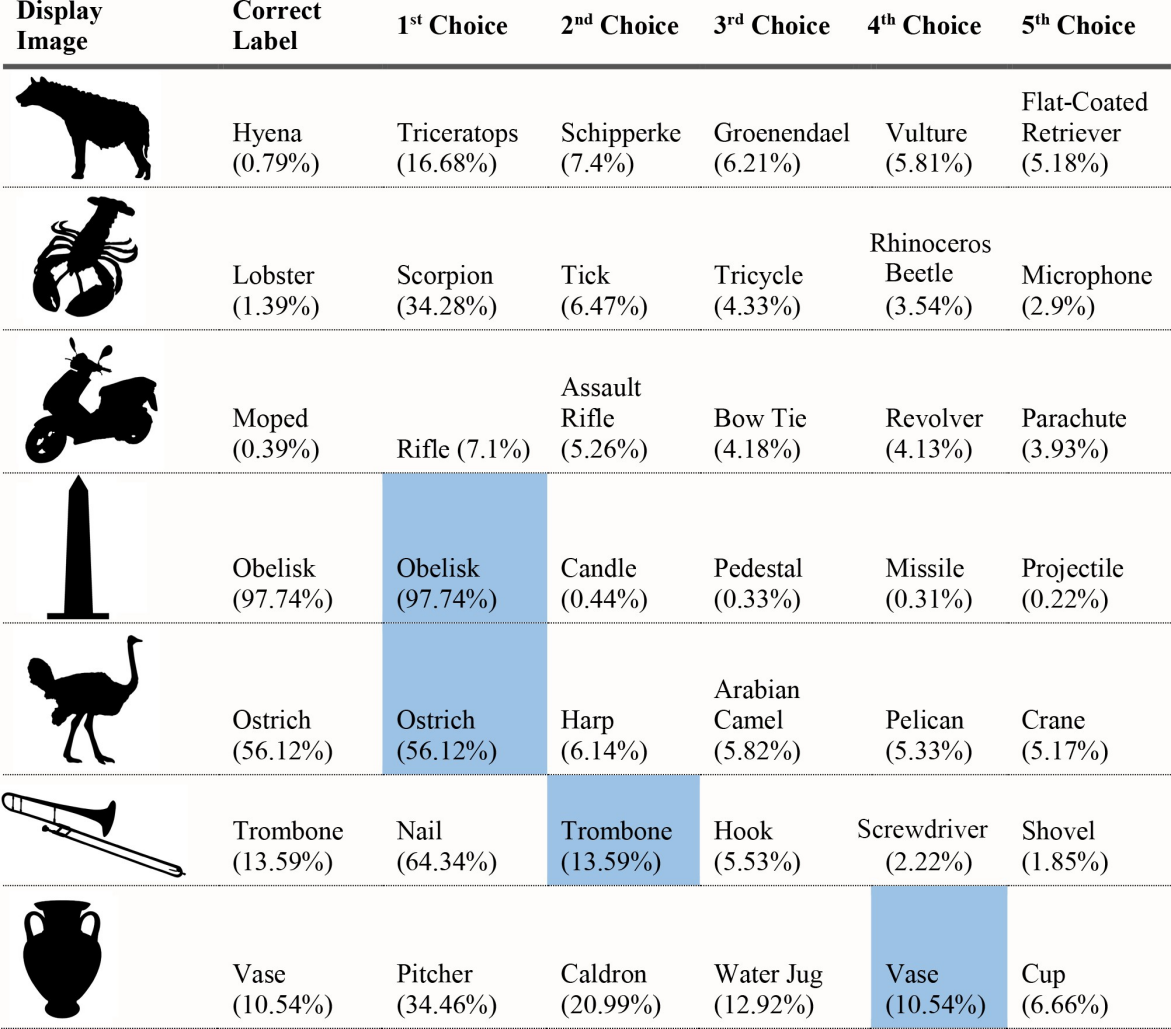
VGG-19 classifications for black object silhouettes Part 4.

For the 40 black silhouettes on a white background, VGG-19 and AlexNet correctly classified 20 and 15 of the 40 presented images (in their top-five classifications), respectively. Figs 19–22 shows the results for VGG-19. Performance was worse for images with white figures on black grounds, where the network classified seven of the 40 images correctly, and for images with red figures on white grounds, where the network classified nine of the 40 images correctly.

#### Experiment 3 Discussion

The results from Experiment 4 are largely consistent with the findings reported by Kubilius et al. In the absence of other information, deep convolutional networks could classify object silhouettes about 50% of the time. In the better-performing VGG-19, there were 12 out of 40 correct top choices, and another eight correct choices in the network’s top-five selections. Classification accuracy was once again higher for artifacts (13/20 correct) than for animals (7/20 correct).

Performance was notably worse for white-on-black and red-on-white figures than for black-on-white figures. One reason for this might be that there are more canonically black objects in the training set than white or red. For example, the cannon is correctly classified when presented as a black figure, but incorrectly classified when presented as a red figure. Instead, “fire engine” appears in the network’s top-five choices, a selection obviously driven by surface properties. Another reason networks might be better at classifying black figures is that they more closely resemble photographic images that were used in network training. Objects will appear very dark or even black if they are between the camera and a bright light, as in actual silhouettes. Possibly, exposure to training examples like these makes the network more likely to accept dark figures as instances of an object, even one that is not canonically dark. The differences in network performance across these three testing sets points to the strong influence of surface information in classification. For humans, a homogenous surface texture would likely not be considered at all in recognition, as the visual system would recognize that there is not enough surface information present to be diagnostic. The network makes no such evaluation and remains highly sensitive to such cues.

Regarding shape, this experiment showed a clear contribution of contour properties in classification of object silhouettes. Within a given display set, all of the test displays shared the same coloration; therefore, all differences in classification responses from the DCNNs involved contour information. Performance at this level demands explanations that go beyond a simple conclusion that DCNNs do or do not process object shape. If DCNNs had access to global shape information, we might have expected their performance to be similar to humans, readily producing accurate classifications for all displays. Indeed, in about 50% of cases, responses seemed to be correct or close in shape to the target object. Still, the results fall far below 100%, even when scored correct if a network generated a correct classification among its top five outputs. The results also contain some rather conspicuous failures to process overall shape. For the porcupine display, for example, VGG-19 gave, as its top choice, "bald eagle". For "lion", AlexNet's top choice was "goose", with high confidence, more than twice the probability of any other response. These results contain important information regarding processing of overall shape. For humans, at least, the lion display is both recognizable as having the shape of a lion and is clearly not shaped at all like a goose. It could be pointed out that failure to give a certain label may merely indicate that the particular shape captured in silhouette may have had a particular vantage point that was uncharacteristic of examples in the training set. The implications for global shape processing here, however, hinge less on the selection of the correct name than on the incorrect answers furnished. “Bald eagle”, as well as runners up “vulture”, “ostrich”, and “buckeye” are not a close shape match for any porcupine.

These results suggest that overall shape is elusive in DCNN responses, but also that something relating to shape allows success in some of the cases tested. Perhaps a deeper analysis of what is meant by shape is needed to understand both the successes and failures of DCNNs. We consider this in successive steps below.

First, why is classification so much better for object silhouettes than for glass figures and shape outlines? We have already commented that the ability to use outlines as depicting shape, although significant in human perception, is not a necessary condition for a DCNN to be shape processor. To confirm that the difference between network performance on silhouettes and outlines was not item specific, we sampled the outline of the 40 silhouettes and tested the network on outline images of the stimuli in Experiment 4. The results closely matched those reported in Experiment 3—the network only classified three of the 40 images in its top-five selections.

What about the better performance of black silhouettes over glass objects? One reason may be that silhouettes successfully reduce distracting texture information, i.e., texture information that would tend to promote a classification other than the correct shape-based response. Glass objects may have contained more misleading surface information than black silhouettes. There are more objects with glass or other transparent or reflective surfaces in the 1000 categories on which the networks were trained than there are uniformly black objects. As mentioned, silhouettes also have the advantage of potentially looking similar to some photographs of the objects during network training if the photographs were taken at sunset or with a bright light behind the object. So, although we still see some misclassifications based on object color, such as “mortarboard” and “academic gown”, the network appears to be overall more robust to black surface textures.

Another important factor contributing to the differences in the previous three experiments is the involvement of figure-ground segmentation. Silhouettes are likely easier to classify than glass objects because the surface information provided contains *no internal contours*. Although figure-ground segmentation is an important part of human shape processing, it is likely that DCNNs produce object classifications with natural images without performing any explicit figure-ground segmentation. In human perception, bounding contours are defining of shape, and shape descriptions are conferred based on the bounding contours of segmented objects [38]. Without any figure-ground segmentation mechanism, all contours in training examples probably have equal status. Nothing designates a bounding contour relevant to overall shape, as opposed to contour information that may be part of surface texture, or noise, etc.

With displays stripped of all contour information except for bounding contours, the networks do better. This suggests that the networks must have used some information about the forms of objects to achieve the performance observed in Experiment 4, even though performance with silhouettes still falls far short of classification that humans readily do with shape. By comparison with the earlier experiments, it also suggests that important (bounding) contour information is more influential when no other contours are present.

What use is made of contour information? An important insight may be provided by the results for the black bear silhouette. The silhouette is substantially simplified such that key points of concavity along a bear outline are connected, mostly by straight lines. This is similar to a classic demonstration in object recognition, Attneave’s cat [39]. Attneave observed that humans can robustly recognize objects whose contour has been changed significantly at a local level, provided that at the global level the spatial relationships between important points along the contour are preserved. Deep networks do not appear to have the same capabilities. We suspect that they are doing essentially the reverse of humans with regard to global and local aspects of shape. We refer to this as the *local contour feature hypothesis*. The top five responses given by the bear silhouette appears to support this conjecture. The silhouette is confused not with other quadrupedal mammals, but with manufactured objects like “warplane”, “stretcher” and “studio couch”, suggesting that the network is using shape information, but at a local, not global level. Locally, there are no parts of a bear’s contour that are straight edges, so the network does not consider “black bear” a probable response, whereas an object like a warplane, though its global shape differs completely from the presented stimulus, has a similar set of local contour segments—straight edges and a few shorter rounded edges. Likewise, the network misclassifies the electric guitar as “shovel”, “hatchet”, or “assault rifle”. No human observer would make such errors, but it is easy to see how local curvature information might produce such responses if global form is disregarded.

The local contour feature hypothesis can also help clarify some positive results from Experiments 1–3. In Experiment 1, we found that deep networks use shape much more when classifying artifacts than when classifying animals. This was also observed in Experiment 4, where 13 of the 20 correctly identified objects were manmade. Artifacts will tend to be discriminable based on local features like curvature more often than animals because they are functional and have different component parts depending on their intended purpose. The hook of a hammer, for example, is highly diagnostic in discriminating it from other shafted tools, as is the curve of a French horn, or the long, thin tubes of a trombone. On the other hand, the local features of animals tend more often to be very similar to each other, with some exceptions such as the horn of a ram, or the legs of a flamingo. Two animals will tend to be more discriminable from each other based on global features, such as the ratio of neck length to body length, or ear size to head size.

In Experiment 2, the only figurines correctly classified were the piano and the great white shark. The network’s successful classification of the piano could be attributable to recognition of local contour features. In particular, the curvature of the top board of a grand piano is highly regular and could be driving the network towards that classification. On the other hand, the curvature of the top board was visible in two of the five additional grand piano figurines we tested on, and the network failed to classify these. For the great white shark, the local contour feature hypothesis fits well with the network’s pattern of response. “Warplane”, “hammerhead”, and “airliner” were all assigned higher probability than the correct object label for the shark figurine. These objects all have some local shape features in common. The fin and tail of a shark are fairly similar to the wings and tail of a plane, but the two objects are hardly confusable to humans. This is because humans group the shape features into a unified whole, while deep networks appear to be influenced by local feature aspects, but perhaps less so their relations to the whole.

In Experiment 3, the beer bottle and coffee mug were classified as “wine bottle” and “cup”. These were scored as correct, since the global shape of the objects are quite similar. Importantly, though, their local curvatures are also quite similar. In particular, the transition from the body to the neck of the beer bottle, and the handle of the coffee mug could be important local shape cues driving the network’s good performance. This seems all the more likely when one considers that “sunglass” is the network’s top classification for the coffee mug. Sunglasses share little global shape similarity with a coffee mug, but the curvature of a sunglass’s frame is often locally quite similar to a cup handle. Likewise, for the tandem bike and trombone, which were not correctly classified, but were assigned higher than chance probability, local contour features like thin straight lines for the trombone and constant curvature in the wheels for the bike could be driving classification. We directly test this hypothesis in Experiments 5.

### Experiment 5

In Experiments 2–4, only the silhouette condition provides some supportive evidence that DCNNs use shape. However, further analysis of the networks’ classification of individual items in Experiments 2–4 suggest that some accurate classification decision is likely based on local contour features, not global object shape. We tested this hypothesis in Experiment 5, by comparing the effects of changing local and global features on network classification performance.

### Experiment 5a

In Experiment 5a, we explicitly tested the hypothesis that deep networks use local shape features, such as the curvature of contour segments, but not global shape, in their classification decisions. We found new examples of shape silhouettes that could be correctly classified and tested to see if the network can still classify them despite changes to their global contour. Preserving most local curvatures, we scrambled the shapes so that the overall shape was radically changed. If the network was robust to these alterations, that would provide evidence that the network is using local shape primitives as cues, rather than the shape’s global contour. If the network utilizes global shape, these disruptions should impede accurate classification.

#### Experiment 5a Method

**Fig 23:**
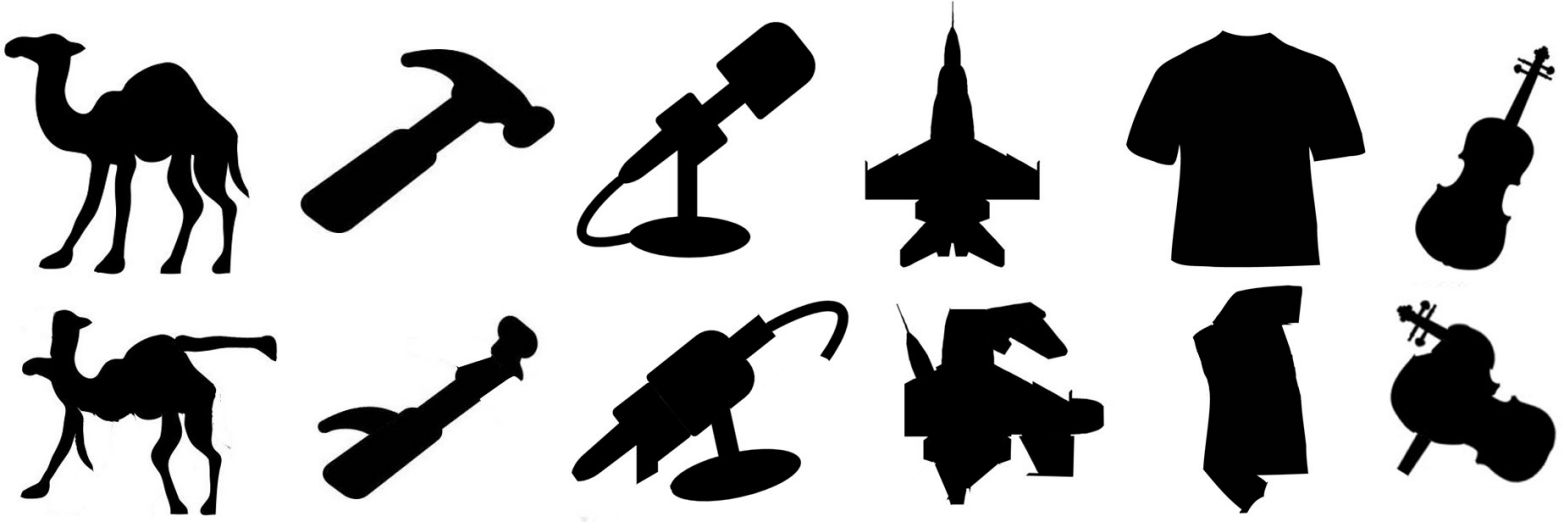
Stimuli used in Experiment 5a. Top row: the original silhouette images, all correctly classified by VGG-19 (appearing in top-five). Bottom row: Scrambled images on which the network was tested.

*Images*. Six images were presented, two from the silhouette database that had been correctly classified, plus four new object silhouettes whose correct label appeared in the network’s top-five selections. Parts of the object were rearranged so that the global contour no longer matched the correct object label, but local edges were preserved. Fig 23 shows the stimulus objects before and after part-scrambling.

*Network*. The images were tested with VGG-19. We omitted simulations on AlexNet, since performance was reliably better in VGG-19 in Experiments 2–4.

*Tests on human subjects*. In addition to evaluating the DCNN’s performance on the six scrambled object silhouettes, we tested human subjects to see if they could recognize the objects after scrambling.

*Participants*. Ten human subjects (two male, eight female, M_age_ = 19.5) were recruited from the University of California, Los Angeles and participated in the study for course credit.

*Design and procedure*. Subjects completed two experiments. For the first experiment, on each trial, subjects were shown one of 30 silhouettes (24 different, unscrambled objects and the six part-scrambled objects), for one second. After presentation of each silhouette, subjects were asked to write down what they were shown on a piece of paper, after which they were free to continue to the next trial.

The second experiment was identical to the first, except that subjects’ exposure duration to each silhouette image was no longer limited to one second. Subjects could view the silhouette for as much time as they wanted before writing down what they believed the object to be on a piece of paper. Once they had recorded their response, they were free to continue to the next trial. As in the first experiment, the second experiment ended when all 30 silhouettes had been presented.

#### Experiment 5a results

**Fig 24:**
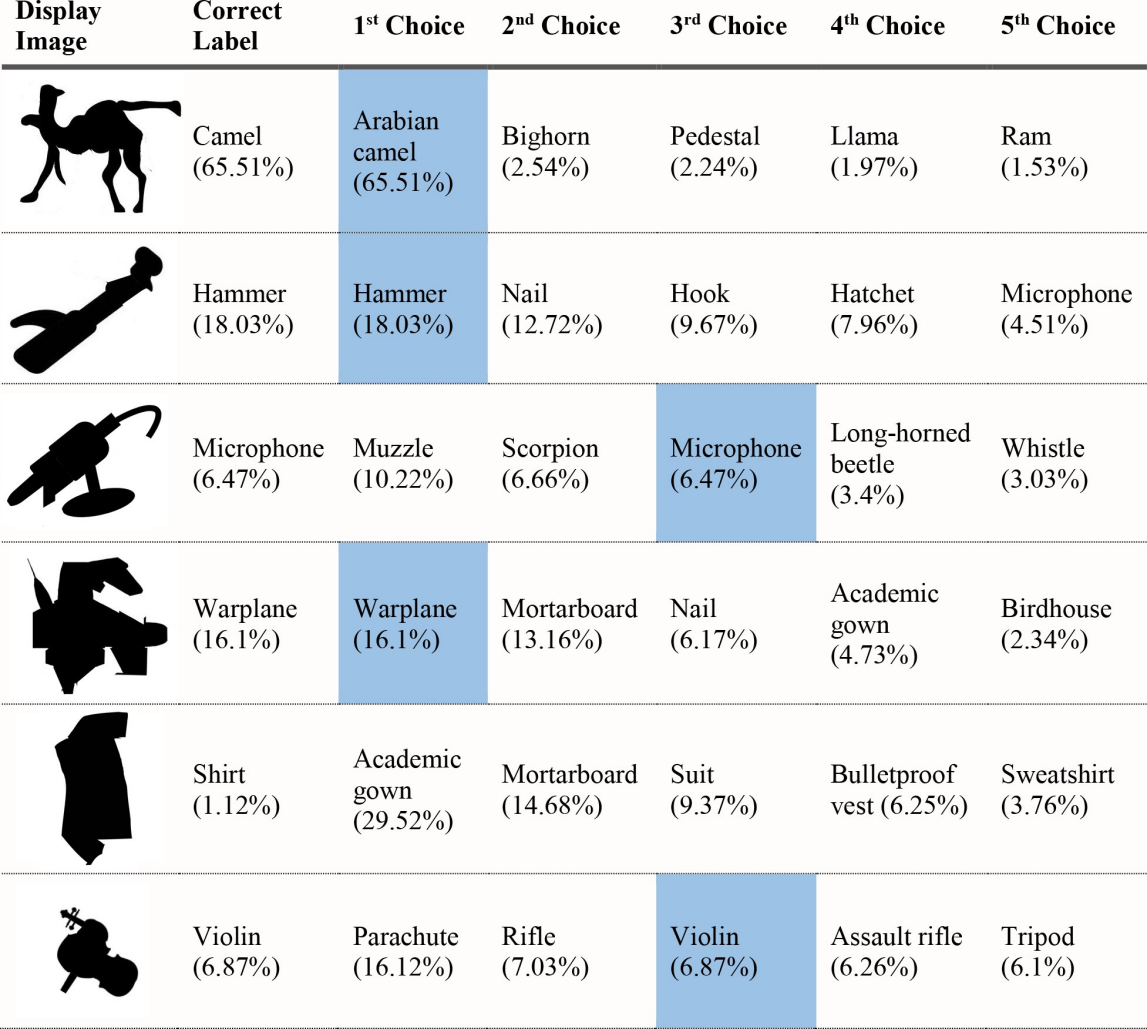
VGG-19 classifications for part-scrambled silhouettes. The leftmost column shows the image presented to the DCNN. The second column shows the correct object label and the classification probability produced by the network for that label. The other five columns show probabilities for the network’s top five classifications, ordered left to right from highest to lowest. Correct classifications are shaded in blue.

The top five responses and associated probabilities for part-scrambled objects are shown in Fig 24. Five of the six scrambled objects were “correctly” classified in the network’s top-five selections. The one correct object label that did not fall in the top-five was “shirt”, but “sweatshirt” did, and the misclassifications made by the network—“suit”, “bulletproof vest”, and “sweatshirt”, for example—are clearly influenced by similar local edge features.

**Fig 25:**
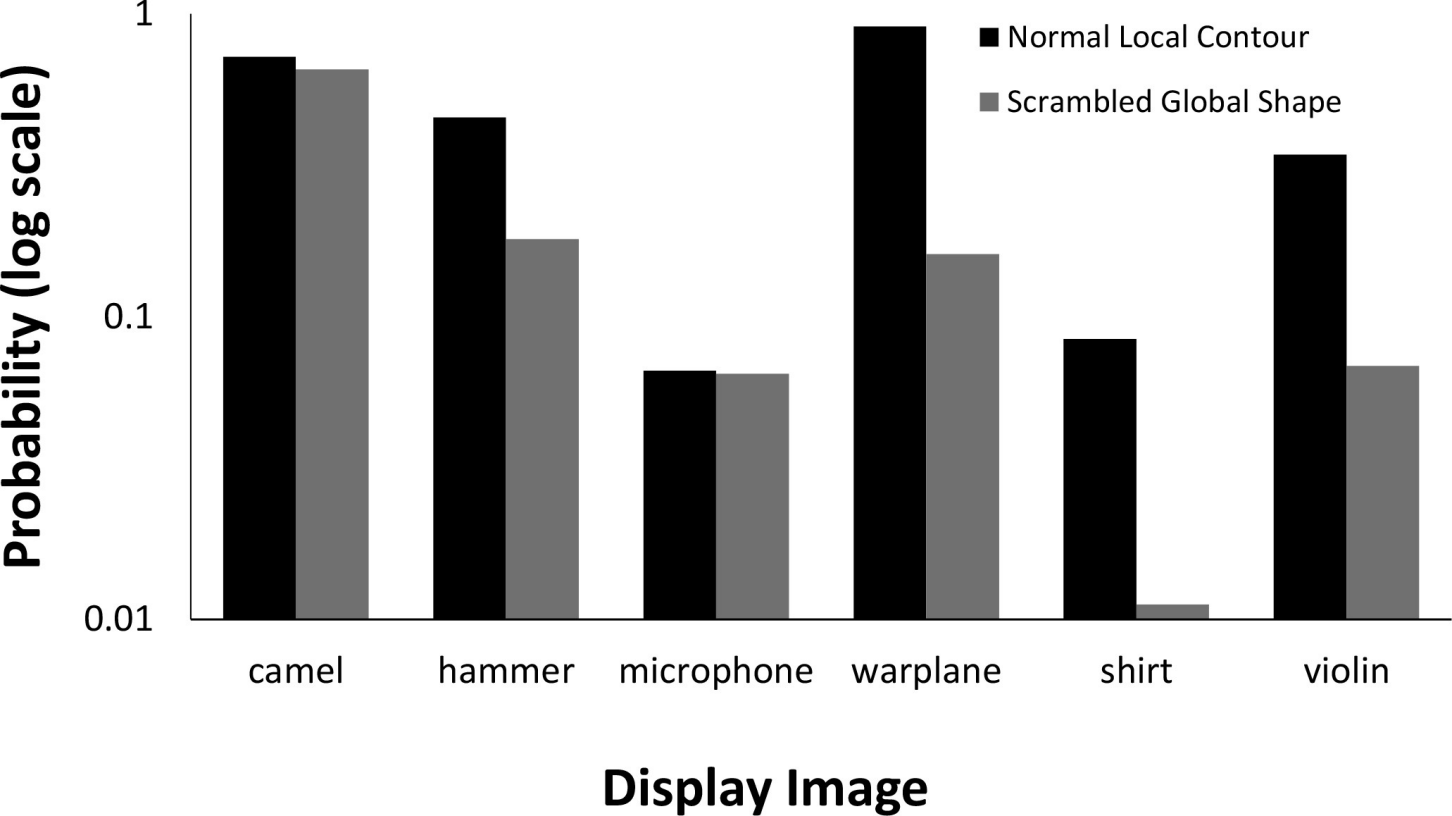
VGG-19 for unscrambled and part-scrambled images. Bars show probabilities for correct responses for each of the objects. Probability is plotted on a logarithmic scale to make small values visible.

We also compared the probability associated with the correct response for the unscrambled image silhouettes with the probabilities for the scrambled silhouettes. Results are shown in Fig 25. On average, the correct response was given a probability 2.26 times higher for unscrambled images than for scrambled images.

*Human object recognition*. Among human participants, subjects correctly identified 23.33% of the part-scrambled objects and 96.67% of the unscrambled objects when their viewing time was restricted to one second. When viewing time was unrestricted, subjects correctly identified 36.67% of part-scrambled objects, and 94.8% of unscrambled objects. Table 1 shows subject accuracy for each of the individual part-scrambled objects.

**Table 1:** Human observers’ performance on individual items for part-scrambled objects.

	Proportion Correct Classification	
Part-Scrambled Object	Display Time: 1 sec	Display Time: Unlimited
Camel	40%	60%
Hammer	10%	0%
Microphone	40%	30%
Warplane	20%	20%
Shirt	20%	30%
Violin	40%	80%

### Experiment 5b

Experiment 5a tested deep networks’ ability to classify objects whose global shape was destroyed, with local curvature largely preserved. We hypothesized that if DCNNs did not classify based on global shape, performance would remain good, provided that enough local contour features were preserved in the scrambled objects. In Experiment 5b, we conducted a complementary study to test the hypothesis that if local contour features are disrupted, but global shape is preserved, network classification accuracy will suffer.

#### Experiment 5b Method

**Fig 26:**
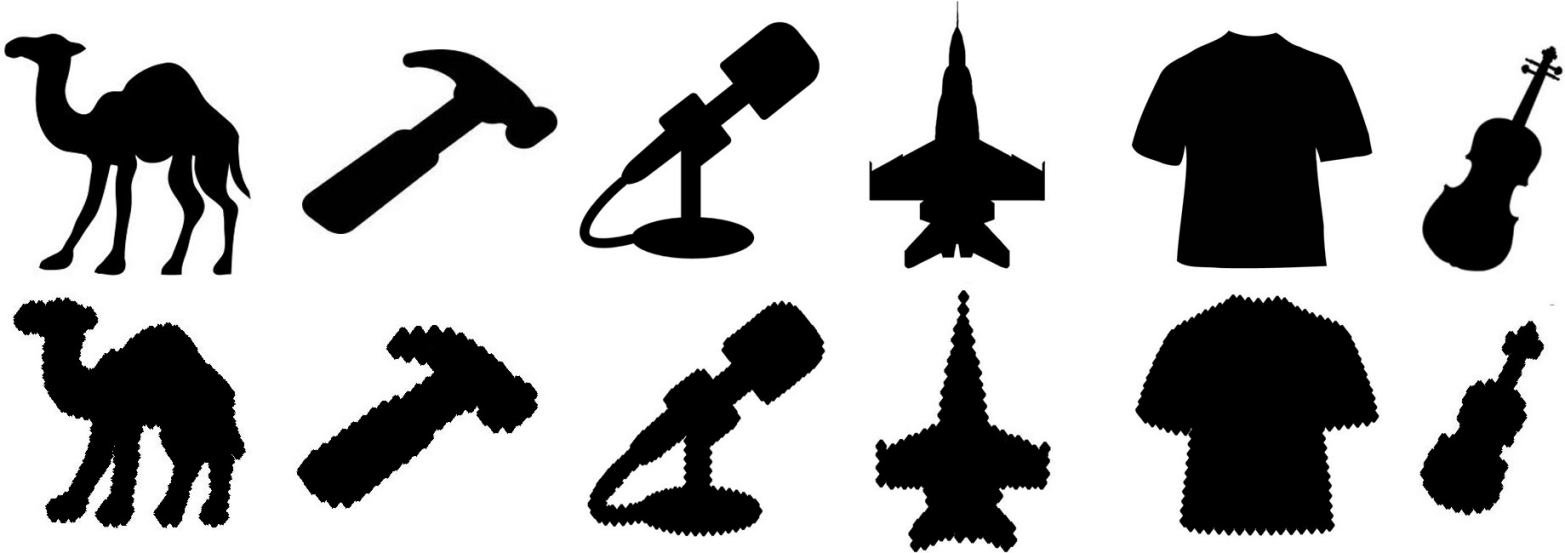
Stimuli used in Experiment 5b. Top row: the original silhouette images, all correctly classified by the network. Bottom row: images with local contour features disrupted.

*Images*. We used the same six silhouette images as in Exp. 5a. In their original format, VGG-19 correctly classified all six images. We disrupted the local contour features in each image by adding jagged edges to the bounding contour, creating a sawtooth effect. Fig 26 shows the six original images and the sawtooth images.

*Network*. The perturbed contour images were tested on VGG-19.

*Test on human subjects*. As in Experiment 5a, we tested humans’ ability to recognize images with global shape preserved and local contour information disrupted.

*Participants*. Ten participants (four male, six female, M_age_ = 20.0) were recruited from the University of California, Los Angeles and participated in the study for course credit. None of the subjects who participated in Exp. 5a participated in this experiment.

*Design and procedure*. The design for the study was identical to Experiment 5a in all respects except for the six disrupted stimuli that were presented. On those trials, subjects were shown the locally disrupted sawtooth images instead of the globally disrupted scrambled images.

#### Experiment 5b results

**Fig 27:**
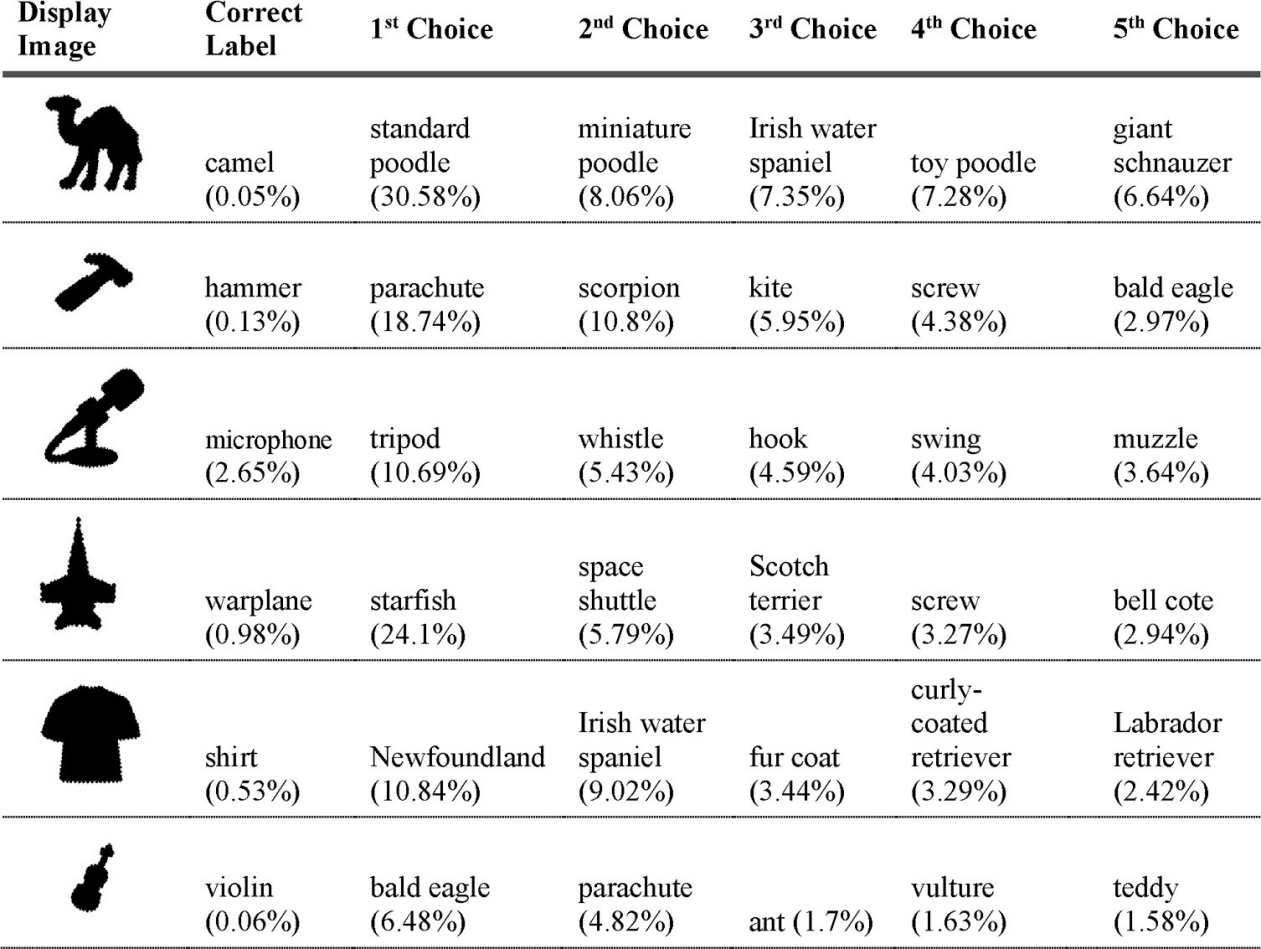
VGG-19 classifications for serrated edge silhouettes. The leftmost column shows the image presented to the DCNN. The second column shows the correct object label and the classification probability produced by the network for that label. The other five columns show probabilities for the network’s top five classifications, ordered left to right from highest to lowest. Correct classifications are shaded in blue.

The network’s top-five classification selections for each of the locally disrupted images are shown in Fig 27. None of the images were correctly classified, either as the first choice or anywhere among the network's top 5 choices, and the average rank of the correct label for perturbed contour images was 96.3.

**Fig 28:**
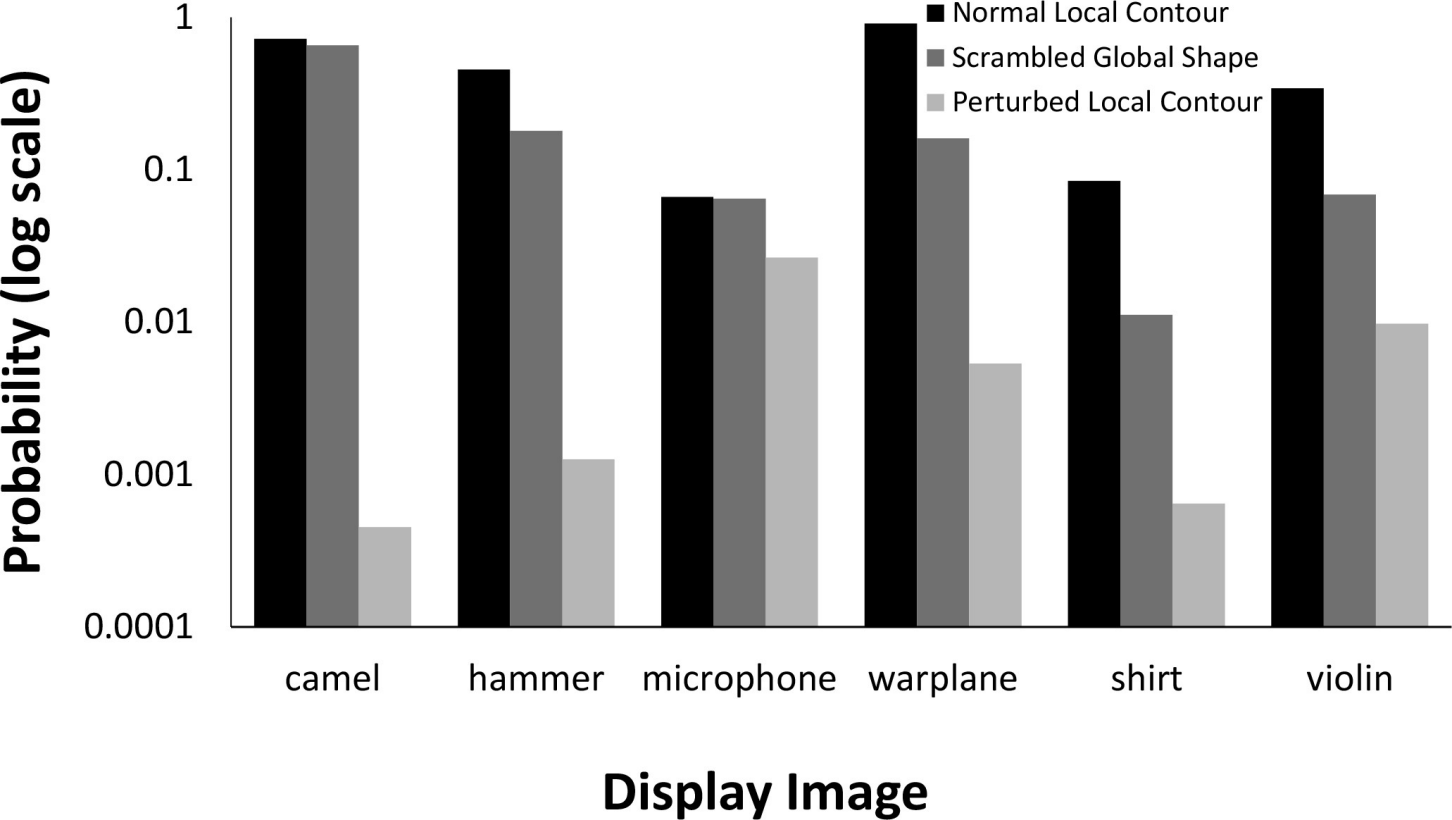
Comparison of VGG-19 performance for locally perturbed contours with unscrambled and part-scrambled images. Bars show probabilities for correct responses for each of the objects. Probability is plotted on a logarithmic scale to make small values visible.

The network performed much worse on images where local contour was disrupted but global shape preserved than with either unperturbed or globally scrambled images. The average probability assigned to shape labels was 58.73 times higher for unperturbed local contour images than for perturbed local contour images, and 26.0 times higher in globally scrambled images than for perturbed local contour images. Fig 28 shows a comparison between these three conditions.

*Human object recognition*. Human participants recognized objects very accurately when local contours were perturbed. When viewing time was limited to one second, subjects’ recognition accuracy on locally disrupted contour silhouettes was 91.67%. With unlimited viewing time, subjects’ accuracy was 96.67%. Subjects’ accuracy on individual items is shown in Table 2. These results did not significantly differ from subjects’ performance on unperturbed object either for the brief presentation condition, *t*(18) = 0.98, *p* = .341, or for the unlimited presentation condition, *t*(18) = 0.82, *p* = .425.

**Table 2:** Human observers’ performance on individual items for perturbed-contour objects.

	Proportion Correct Classification	
Locally Perturbed Object	Display Time: 1 sec	Display Time: Unlimited
Camel	100%	100%
Hammer	100%	100%
Microphone	90%	100%
Warplane	90%	90%
Shirt	100%	100%
Violin	70%	90%

#### Experiment 5 Discussion

The results of Experiments 5a and 5b suggest robust and interesting differences between humans and DCNNs in the use of shape information. Humans mostly failed to correctly recognize the objects presented in Experiment 5a. For one or two of the objects, they saw some recognizable parts (e.g., for the scrambled violin, one subject said “I see some pieces of a broken guitar”). Even where some parts could be recognized, every human observer pointed out that the scrambled objects are not actual versions of the label that would be correct for the unscrambled versions. In contrast, when human subjects were shown objects whose global shape was preserved, but whose local contour features were changed, they performed extremely well on the recognition task, both with short exposures and unlimited viewing time.

The DCNN results showed the reverse pattern of the human results. For VGG-19, classification for objects whose global shape had been destroyed by local scrambling remained very strong. However, in the local contour disruption condition, the network’s classification accuracy fell off dramatically. Despite preservation of global shape that allowed essentially perfect human object classification for both brief exposures and unlimited viewing, VGG-19 did not select any correct label for any object in Exp. 5b among its top five choices. The influence of local contour information is clearly visible in many of the network’s top-five selections from this simulation. Animals like poodles, curly-coated retrievers, and Scotch terriers are often selected despite no similarity in global shape, likely because the local features resemble a curly-furred animal. These results suggest that the network’s processing of shape is restricted to relatively local features along an object’s bounding contour, but not the shape as a whole. They also represent a clear difference between human and deep network recognition processes. Whereas local contour features seem to play a key role in deep network classification, global shape seems to have primacy in human perception.

These results, along with those of the earlier experiments, push us to think about what is really meant by shape. The convolution operations that form the groundwork for DCNNs are certainly capable of responding to local oriented contrast, as such filters have long been used to model orientation sensitivity [35]. For a shape feature such as the “claw” part of the hammer image, it would be sufficient for the network to encode a few orientations in proximity and in a certain spatial relation. These are, undoubtedly, aspects of “shape”. And our results suggest that they are accessible within the trained AlexNet and VGG-19 networks. There are other, more global, notions of shape, however. Larger relations of parts, and overall characteristics, such as aspect ratios, may generally be inaccessible. The network was remarkably undeterred by serious scrambling of parts, despite the fact that this scrambling undoubtedly also disrupted some local features of the sort we have noted here.

The issue of local shape features vs. global shape descriptions bears an important relation to questions of subsymbolic and symbolic representations in visual perception [36]. A number of phenomena indicate that human vision produces abstract shape representations that capture the gist of objects and support similarity relationships, despite local noise, and variations in local elements [40]. Even the perception of a clear object edge or the notion of a continuous contour are abstractions from earlier inputs of local oriented contrast in various locations and different spatial frequencies in the same location (for discussion, see [41]). Conversely, measurement of oriented contrast in two nearby locations and use of some conjunction of orientations in nearby positions can be done without conversion to a symbolic representation. These are the beginnings of shape descriptions, or perhaps even local shape descriptions. Deep networks may be accessing shape features that are conjunctions of local orientations and their relations; indeed, such representations may lie somewhere in the transition between subsymbolic and symbolic representations of an object’s shape (see [42] for a related proposal regarding the representation of contour shape). Taken together with the results of Experiments 1–4, the findings from Experiment 5 are consistent with the idea that DCNNs access aspects of local edge and curvature information, but not a representation of how local parts spatially relate to each other as a whole.

## Discussion

The purpose of this work was to determine the extent to which shape information is represented and used for recognition in trained deep convolutional networks. We tested deep networks trained on ImageNet to determine if shape information is relevant at all to DCNN object recognition performance, and if it is, how shape cues are weighted compared to other information, such as texture and context.

In Experiment 1, we tested VGG-19 for sensitivity to shape apart from appropriate texture and surface information, showing the network 40 object silhouettes with a different object’s surface texture overlaid on each shape. Evidence for use of shape information was weak: The correct label based on shape was chosen as the first choice classification for only 5 of the 40 objects sampled, and the average rank among network outputs for the correct shape was 209. Where evidence of shape influences on classification did appear, however, they seemed to depend highly on the kind of object being classified. While classification of artifacts and rigid objects appeared to depend on shape more than on texture cues, the opposite was true for images of animals. Even for artifacts in which the network weights shape more strongly, the absence of typical surface texture greatly reduced the network’s classification accuracy, resulting in many spurious classifications. The importance of texture information in object recognition was also seen in Experiment 2, where glass objects with no shape similarity to the presented stimuli were selected preferentially over the object whose shape matched the glass figurine, and in Experiment 4, where performance changed dramatically depending on whether a silhouette was black, white, or red.These results differ greatly from what is observed in research on human vision. Several studies (e.g., [43, 44, 28–33]) have found that texture plays little role in facilitating human object recognition.

In Experiments 2 and 3, we presented two networks with images whose shape matched object categories that the network had been trained to recognize, while differing in terms of context and surface texture. Deep networks were in general unable to classify glass figurines or object outlines in the absence of other cues that are typically present in natural images. Across both datasets, only four of 80 presented images were correctly classified, and analyses of the probabilities assigned to each object label revealed that the network was assigning near-minimum probabilities to the correct objects in all but a few cases.

A possible explanation for the network’s poor performance in Experiment 3 is that the outline images are flat, lacking volumetric cues that might be present in 2D photographs of real objects. If the network depends on volumetric cues to recognize objects, the flatness of the outline images might prevent it from matching these images to its trained categories. This is an intriguing hypothesis, but the network was no better for glass figurines than for object outlines, even though figurines have 3D structures that would be quite similar to those of objects the network is trained to recognize. Moreover, the network did comparatively well in Experiment 4 classifying object silhouettes, which also lack any volumetric properties.

Experiment 4 found some evidence for use of shape information in object recognition by deep networks. Networks were presented with object silhouettes and correctly classified 20 of the 40 images (based on the top-five criterion), as well as assigning non-minimum probabilities to several others. One explanation for the networks’ superior performance for silhouettes is that these displays more fully eliminate competing surface texture information than do objects with glass surfaces, or objects whose texture exactly matched the background. They also remove orientation information that is not part of an object's bounding contour, thus preserving the orientation information most likely to be diagnostic of an object's shape. This selective preservation of orientation is probably especially helpful, as it seems unlikely that DCNNs trained for object classification have any differential representation of a bounding contour vs. any other contour.

Classification of object silhouettes would not be possible without some edge-based recognition capabilities, but it is unlikely that deep networks accessed global form for the task. Instead, the network appears to be to have extracted contour segments and local features based on some relations of proximate contour orientations. Indirect evidence supporting this explanation appeared in some of the network’s misclassifications. For example, a great white shark figurine had warplane and airliner in its top-five labels, likely due to the local similarity between a shark’s fins and a plane’s wings. Likewise, a porcupine was misclassified as a bird, probably because its spines had features in common with feathers, although globally birds and porcupines differ greatly.

Experiment 5 sought direct evidence for the local contour feature hypothesis. In Experiment 5a we used silhouette images, including some of the best performing ones from Experiment 4, for which the networks produced accurate classification outcomes. These images were then scrambled in such a way that curvature of local edge segments was preserved, but the shape as a whole was radically altered. Networks performed nearly as well on these scrambled objects as they did on the original images. On the other hand, in Experiment 5b, when global shape was preserved but local contour features were changed by adding jagged edges, network classification became extremely poor. These results suggest that the features relating to shape that figure in recognition in deep networks relate to curvature or orientation relations of highly local parts of the contour. The network recognizes the object by the mere presence of these features, not by how they might globally relate to each other in space. The convolution operations that occur at the earliest levels of DCNNs are well suited to extract local oriented contrast, and conjunctions of nearby orientations could serve as a serviceable marker for local curvature, as well as local shape features. This is why rearranging existing parts of a silhouette has little effect on network performance (since local feature extraction is the same) while the addition of a serrated border to the silhouette destroys network performance. The opposite is true in human perception.

In human vision, the transition from subsymbolic to symbolic processing depends critically on how encoded features fit together into a unified whole [36, 45]. Identifying the curvature of contour segments may be an important first step in this transition, but it cannot account for the robust capabilities of our visual system to classify objects based on their shape. For example, Attneave [39], showed that all local curvature segments in a cat can be straightened without detriment to its recognizability. We actually tested one image, a bear, that had straightened segments, much like Attneave's cat, in Experiments 1 and 4. The probabilities assigned by the network were less than chance with both inconsistent texture, in Exp. 1 (.04%), and in silhouette form, in Exp. 4 (.03%). Consistent with these ideas, the bear image in both cases was unequivocally seen as a bear by human observers. This and many other phenomena of human perception suggest that relationships between contour segments and object parts are central to human object recognition and shape perception, more so than encoding local features.

In the introduction, we mentioned three levels of questions that might be asked regarding DCNNs and the use of shape information. The present results apply to the first level: the important question of understanding the role of shape in DCNNs trained to do object recognition. We believe that our results provide a good picture of the use of shape information by trained DCNNs, specifically suggesting a profound difference between the accessibility of local shape features and more global aspects of shape. The results of this study appear to strongly suggest that such models do not classify based on an object’s global shape cues. These results regarding the shape sensitivity of trained networks are important for comparisons of human perception with DCNNs, which in other respects have been argued to mirror the human visual brain and human behavior in a remarkable number of ways.

We noted earlier a second level of question–whether DCNNs can be trained from scratch to classify based on global shape information. Our present results clearly suggest that standard training on the ImageNet database does not produce such capabilities, but it leaves open the question of whether deep networks are *incapable* of classification based on global shape features. The photographs in ImageNet tend to show a single object in the foreground of the image. It is possible that training conditions in which the object is displayed in a more complete scene would produce better shape sensitivity because the network would learn to extract the spatial relationships between objects. Another way the network might be trained to encode global shape would be to include training examples with nondiagnostic texture and local shape properties. The network might have too many other rich cues for classification to develop global shape sensitivity under standard training conditions, but perhaps could encode global shape if deprived of some of these other information streams. Our suspicion is that even under training conditions specifically selected to develop global shape representations, DCNNs will not be able to classify based on global shape alone, but these hyotheses can only be confirmed through experimentation on untrained neural networks.

### Conclusion

In human vision, abstract representations of shape describe how the various parts of an object spatially relate to one another. They are critical for recognition across a variety of viewing conditions, and are robust to perturbations of local contour features. Deep networks have impressive capabilities for object recognition, but they do not appear to handle the problem of recognition the same way humans do. Unlike humans, surface texture appears to be an equally strong cue for recognition as shape. Moreover, the shape information used by by deep networks is highly limited: DCNNs appear to be capable of encoding local shape features including local edge segments and relations. Sensitivity to how these local features fit together as a whole is lacking; DCNNs trained for object recognition do not appear to represent global shape at all.

## Methods

### Ethics statement

All research on human subjects in this and subsequent experiments was under IRB approval (IRB#11-002079-CR-00001).

### Network

Tests were conducted on VGG-19 [27] and AlexNet [4]. For most experiments, the results of the better-performing VGG-19 are reported. Both networks were trained on the ImageNet database prior to testing. Tests were conducted using the Neural Networks Toolbox from Matlab 2017b.

### Materials

Testing images are described in the Methods description for individual experiments. In all experiments, the correct classification for the testing images was among the 1000 object categories that the networks had been trained to classify.

## Supporting information

S1 File Results from outlines sampled from Exp. 4 silhouettes. The leftmost column shows the image presented to VGG-19. The second column from the left shows the correct object label and the classification probability produced for that label. The other five columns show probabilities for the network’s top five classifications, ordered left to right in terms of the probability given by the network. Correct classifications are shaded in blue.
(DOCX)

S2 File URLS For test images. (DOCX)
