# Supplementary material for: Deep convolutional networks do not classify based on global object shape
Source: PLoS Comput Biol. 2018 Dec 7;14(12):e1006613. doi: 10.1371/journal.pcbi.1006613 (PMC6306249; doi:10.1371/journal.pcbi.1006613)
Supplement: S1 File — The leftmost column shows the image presented to VGG-19. The second column from the left shows the correct object label and the classification probability produced for that label. The other five columns show probabilities for the network’s top five classifications, ordered left to right in terms of the probability given by the network. Correct classifications are shaded in blue. (DOCX) [file pcbi.1006613.s001.docx]

**Supplemental Materials—Additional Data**

**Results from Outlines Sampled from Exp. 4 Silhouettes**

| **Display Image** | **Correct Label** | **1^st^ Choice** | **2^nd^ Choice** | **3^rd^ Choice** | **4^th^ Choice** | | **5^th^ Choice** |
| --- | --- | --- | --- | --- | --- | --- | --- |
| 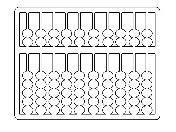 | Abacus (61.71%) | Abacus (61.71%) | Space Bar (15.29%) | Computer Keyboard (10.29%) | | Typewriter Keyboard (8.05%) | Plate Rack (0.86%) |
| 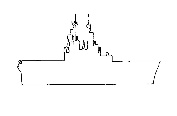 | Aircraft Carrier (0.61%) | Nail (9.32%) | Hook (7.86%) | Syringe (6.26%) | | Warplane (3%) | Stretcher (2.62%) |
| 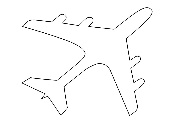 | Airplane (0.02%) | Sunglass (4.61%) | Hammer (3.6%) | Nail (3.15%) | | Hatchet (2.98%) | Envelope (2.87%) |
| 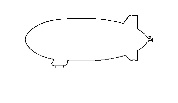 | Airship (0.24%) | Sunglass (4.79%) | Envelope (4.63%) | Bow (3.49%) | | Sunglasses (3.22%) | Hook (2.98%) |
| 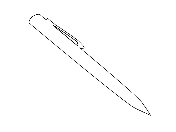 | Ballpoint (8.8%) | Letter Opener (60.32%) | Ballpoint (8.8%) | Screwdriver (4.72%) | | Hair Slide (4.11%) | Safety Pin (2.4%) |
| 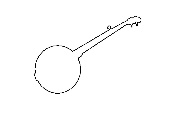 | Banjo (1.25%) | Syringe (34.75%) | Strainer (11.66%) | Ladle (6.68%) | | Microphone (4.79%) | Sunglass (3.18%) |
| 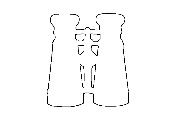 | Binoculars (0.02%) | Beaker (30.97%) | Hook (4.43%) | Safety Pin (4.09%) | | Corkscrew (3.72%) | Pitcher (2.51%) |
| 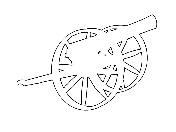 | Cannon (0.06%) | Envelope (9.98%) | Web Site (3.17%) | Hook (2.39%) | | Jersey (2.02%) | Notebook (1.71%) |
| 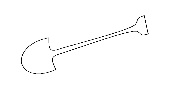 | Shovel (4.59%) | Hook (19.27%) | Microphone (8.37%) | Nail (7.77%) | | Syringe (6.29%) | Ladle (4.91%) |
| 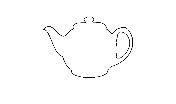 | Teapot (0.03%) | Envelope (15.86%) | Hook (5.74%) | Cleaver (3.04%) | | Sunglass (1.93%) | Sunglasses (1.62%) |
| 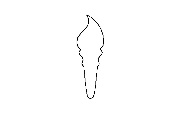 | Torch (0.06%) | Hook (9.36%) | Black Widow (5.11%) | Nail (2.43%) | | Garden Spider (2.4%) | Nematode (2.22%) |
| 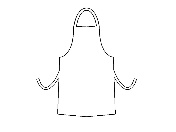 | Apron (0.24%) | Hook (17.17%) | Envelope (14.85%) | Web Site (1.93%) | | Mouse (1.9%) | Microphone (1.84%) |
| 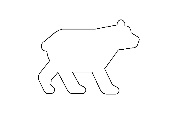 | Bear (0.01%) | Envelope (11.46%) | Hook (2.14%) | Cleaver (1.84%) | | Web Site (1.78%) | Corkscrew (1.66%) |
| 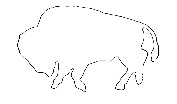 | Bison (0.01%) | Envelope (14.29%) | Nematode (2.24%) | Hook (2.2%) | | Mouse (1.99%) | Fire Screen (1.68%) |
| 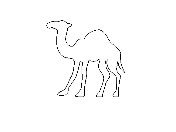 | Camel (0.05%) | Envelope (16.07%) | Hook (2.52%) | Cleaver (2.42%) | | Corkscrew (2.32%) | Web Site (1.73%) |
| 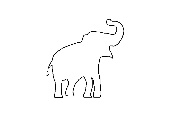 | Elephant (0.02%) | Envelope (9.91%) | Corkscrew (5.23%) | Hook (5.22%) | | Cleaver (4.96%) | Can Opener (2.73%) |
| 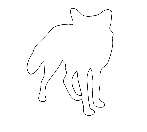 | Fox (0.02%) | Envelope (11.03%) | Hook (3.85%) | Nematode (2.5%) | | Corkscrew (2.09%) | Cleaver (2.02%) |
| 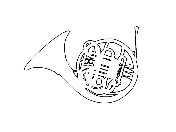 | French Horn (0.62%) | Jersey (7.29%) | Hatchet (6.05%) | Envelope (5.86%) | | Letter Opener (5.46%) | Hook (2.66%) |
| 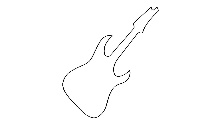 | Guitar (1.53%) | Hook (6.26%) | Envelope (5.72%) | Cleaver (5.05%) | | Bow (3.6%) | Microphone (3.34%) |
| 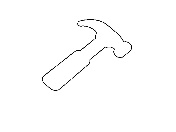 | Hammer (1.67%) | Hook (40.5%) | Can Opener (4.33%) | Corkscrew (3.56%) | | Safety Pin (2.5%) | Cleaver (2.39%) |
| 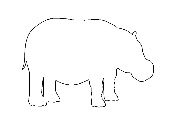 | Hippopotamus (0.01%) | Envelope (8.96%) | Hook (4.2%) | Corkscrew (3.63%) | | Fire Screen (2.27%) | Jigsaw Puzzle (2.11%) |
| 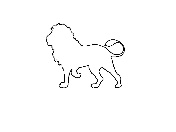 | Lion (0.01%) | Envelope (12.11%) | Hook (5.77%) | Corkscrew (4.42%) | | Cleaver (3.27%) | Letter Opener (2.53%) |
| 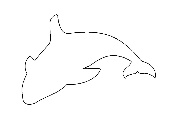 | Orca (0.01%) | Nematode (19.45%) | Hook (11.26%) | Jigsaw Puzzle (5.31%) | | Corkscrew (3.72%) | Envelope (3.34%) |
| 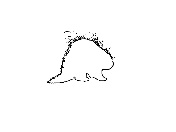 | Porcupine (0.01%) | Envelope (8.76%) | Corkscrew (8.25%) | Parachute (2.84%) | | Letter Opener (2.43%) | Hook (2.29%) |
| 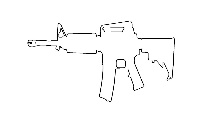 | Rifle (2.21%) | Nail (9.64%) | Hook (4.62%) | Envelope (4.56%) | | Safety Pin (3.63%) | Corkscrew (3.3%) |
| 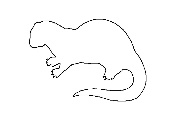 | Otter (0%) | Envelope (11.04%) | Nematode (9.81%) | Hook (9.27%) | | Corkscrew (8.14%) | Letter Opener (3.83%) |
| 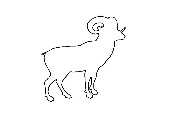 | Ram (0.01%) | Envelope (19.37%) | Hook (3.34%) | Corkscrew (3.19%) | | Letter Opener (2.15%) | Cleaver (1.98%) |
| 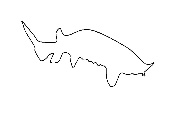 | Sturgeon (0.01%) | Corkscrew (8.5%) | Fire Screen (7.37%) | Envelope (5.11%) | | Nematode (4.98%) | Hook (4.78%) |
| 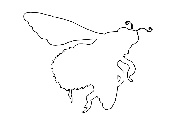 | Bee (0%) | Envelope (23.04%) | Hook (3.25%) | Cleaver (2.95%) | | Corkscrew (2.56%) | Loupe (1.79%) |
| 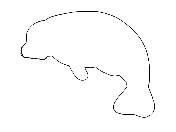 | Dugong (0.01%) | Hook (10.57%) | Nematode (7.2%) | Envelope (7.19%) | | Jigsaw Puzzle (4.2%) | Bow (3.62%) |
| 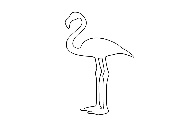 | Flamingo (0.04%) | Hook (13.12%) | Envelope (12.2%) | Corkscrew (6.06%) | | Binder (2.74%) | Nematode (2.44%) |
| 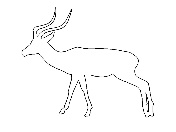 | Gazelle (0.33%) | Nematode (10.43%) | Corkscrew (5.93%) | Fire Screen (5.34%) | | Hook (5.12%) | Envelope (4.46%) |
| 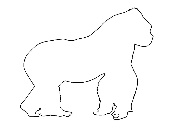 | Gorilla (0%) | Envelope (14.88%) | Jigsaw Puzzle (6.71%) | Fire Screen (3.62%) | | Hook (2.08%) | Nematode (1.94%) |
| 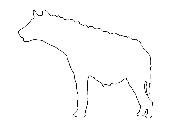 | Hyena (0.03%) | Envelope (7.46%) | Corkscrew (5.3%) | Hook (3.21%) | | Fire Screen (2.15%) | Quill (1.45%) |
| 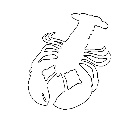 | Lobster (0.08%) | Envelope (26.08%) | Binder (2.82%) | Web Site (2.02%) | | Laptop (1.93%) | Jersey (1.7%) |
| 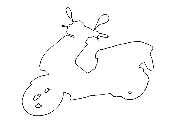 | Moped (0.01%) | Envelope (34.58%) | Jigsaw Puzzle (4.64%) | Hook (3.48%) | | Can Opener (2.82%) | Corkscrew (1.73%) |
| 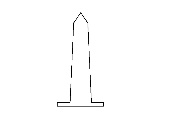 | Obelisk (0.06%) | Syringe (7.45%) | Rule (4.57%) | Nail (3.54%) | | Crane (3.42%) | Wall Clock (3.08%) |
| 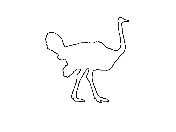 | Ostrich (0.04%) | Hook (10.67%) | Corkscrew (9.78%) | Nematode (6.05%) | | Envelope (4.55%) | Cleaver (3.38%) |
| 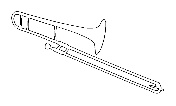 | Trombone (57.87%) | Trombone (57.87%) | Hook (8.79%) | Nail (6.4%) | | Safety Pin (6.34%) | Cornet (3.81%) |
| 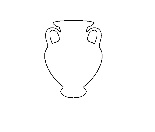 | Vase (0.25%) | Envelope (12.67%) | Cleaver (4.26%) | Red Wine (3.67%) | | Hook (3.61%) | Sunglasses (2.39%) |

*Note*. The leftmost column shows the image presented to VGG-19. The second column from the left shows the correct object label and the classification probability produced for that label. The other five columns show probabilities for the network’s top five classifications, ordered left to right in terms of the probability given by the network**.** Correct classifications are shaded in blue.
