# Supplementary material for: Deep convolutional networks do not classify based on global object shape
Source: PLoS Comput Biol. 2018 Dec 7;14(12):e1006613. doi: 10.1371/journal.pcbi.1006613 (PMC6306249; doi:10.1371/journal.pcbi.1006613)
Supplement: S2 File — (DOCX) [file pcbi.1006613.s002.docx]

**Supplemental Materials- Images Used in Experiments**

**Natural Images**

https://www.shutterstock.com/image-photo/black-bear-alaska-171509846?irgwc=1&utm_medium=Affiliate&utm_campaign=TinEye&utm_source=77643&utm_term=

**Silhouettes**

Bear

http://i.ebayimg.com/00/z/YGcAAMXQVT9S3uwI/%24_1.JPG?set_11.JPG?set_id=807

Elephant

http://image.spreadshirtmedia.com/image-server/v1/designs/10177614%2Cwidth=200%2Cheight=200

Fox

https://publicdomainvectors.org/tn_img/nicubunu_Fox_contour.png

Electric Guitar

https://www.shareicon.net/data/256x256/2015/10/19/658401_music_512x512.png

Hammer

http://clipart-library.com/images/AcbK5pRoi.png

Hippopotamus

<http://naklejki-na-avto.ru/images/product/s/83de0ee.png>

Camel

http://clipartmag.com/images/camel-clipart-16.jpg

Lion

http://images.clipartpanda.com/elephant-clipart-silhouette-silhouette_of_a_lion_the_king_of_the_beasts_0515-1011-2616-2804_SMU.jpg

Orca

https://image.flaticon.com/icons/png/256/47/47332.png

Porcupine

https://3.bp.blogspot.com/-nhQa_z9Msx8/UKksCYOgSPI/AAAAAAAAC58/-EPAkL0qUC4/s1600/Porcupine_%28PSF%29c.png

Moped

https://felallen.files.wordpress.com/2011/03/bike.jpg

Hyena

http://clipground.com/images/hyena-head-silhouette-clipart-17.jpg

Bee

https://www.publicdomainpictures.net/pictures/120000/velka/silhouette-of-a-bumble-bee.jpg

Bison

http://www.spstencils.com/wp-content/uploads/2014/08/Bison.jpg

Sturgeon

https://orig00.deviantart.net/6f07/f/2013/130/7/5/sturgeon_silhouette_by_lamastok-d64quu7.png

Ram

http://moziru.com/images/dall-sheep-clipart-silhouette-2.jpg

Gazelle

https://gallery.yopriceville.com/Free-Clipart-Pictures/Silhouettes-PNG/Gazelle_Silhouette_PNG_Transparent_Clip_Art_Image

Gorilla

http://moziru.com/images/shaow-clipart-gorilla-1.png

Obelisk

https://www.pinterest.com/pin/495747871473179204

Ostrich

http://www.freestencilgallery.com/wp-content/uploads/2016/09/Ostrich-Silhouette-Stencil-thumb.jpg

Lobster

https://i.pinimg.com/originals/9f/7b/f6/9f7bf63b8393eac269187358cef52627.jpg

Flamingo

<http://www.publicdomainpictures.net/pictures/160000/velka/flamingo-silhouette-clipart.jpg>

Manatee

<http://moziru.com/images/manatee-clipart-silhouette-2.jpg>

Sea Otter

<http://moziru.com/images/nutria-clipart-sea-otter-8.gif>

Abacus

<http://sweetclipart.com/multisite/sweetclipart/files/abacus_black.png>

Binoculars

<http://www.silhouettevectorstock.com/blog/wp-content/uploads/2014/07/binocular.jpg>

Aircraft carrier

<http://moziru.com/images/aircraft-carrier-clipart-navy-ship-20.jpg>

Airplane

<https://upload.wikimedia.org/wikipedia/commons/thumb/c/c5/Airplane_silhouette.svg/2000px-Airplane_silhouette.svg.png>

Blimp

<http://www.supercoloring.com/sites/default/files/silhouettes/2015/05/blimp-black-silhouette.svg>

Apron

<https://image.freepik.com/free-icon/apron-silhouette_318-61297.jpg>

Assault Rifle

<http://moziru.com/images/machine-gun-clipart-16.png>

Balloon

<http://stickurz.com/1315-thickbox_default/kid-balloon.jpg>

Banjo

<http://www.signtorch.com/vector/super/art/music/ss/banjo2a__ss.gif>

Ballpoint pen

<https://cdn.pixabay.com/photo/2016/04/09/16/09/pen-1318344_1280.png>

Cannon

<http://www.publicdomainpictures.net/pictures/140000/velka/cannon-1445555048WIF.jpg>

French horn

<http://images.all-free-download.com/images/graphiclarge/french_horn_silhouette_clip_art_12349.jpg>

Shovel

<http://www.clker.com/cliparts/i/i/f/d/x/y/garden-shovel-hi.png>

Teapot

<http://clipart-library.com/images/rTjKAr4gc.png>

Torch

[http://silhouettegarden.com/files/images//torch-silhouette-thumbnail.png](http://silhouettegarden.com/files/images/torch-silhouette-thumbnail.png)

Trombone

<https://cdn.pixabay.com/photo/2012/04/13/00/41/trombone-31379_1280.png>

Vase

<http://www.supercoloring.com/sites/default/files/silhouettes/2015/05/greek-vase-black-silhouette.svg>

**Textures**

<https://thumbs.dreamstime.com/b/textura-de-los-tableros-de-madera-del-barril-94519935.jpg>

<https://www.colourbox.com/preview/3648389-broom-texture.jpg>

<https://previews.123rf.com/images/oleksiy/oleksiy1202/oleksiy120200005/12344085-cuirass-seamless-texture-background--Stock-Photo.jpg>

<https://static1.squarespace.com/static/573f61b920c647f6635d33ca/t/57b1f54f15d5db793ce060c8/1471280469537/CloseUpGolfBall.jpg?format=1500w>

<https://st.depositphotos.com/1177973/3545/i/450/depositphotos_35451975-stock-photo-yellow-beautiful-honeycomb-with-honey.jpg>

<http://www.dyerkickers.org/wp-content/uploads/sites/677/2017/05/header.jpg>

<http://www.clker.com/cliparts/b/f/2/e/1452375469638842217stone-wall-texture.jpg>

<https://www.photohdx.com/images/2015/11/red-velvet-texture-background.jpg>

<https://image.shutterstock.com/z/stock-photo-guacamole-a-seamless-food-texture-use-this-texture-in-fabric-and-material-printing-image-584985802.jpg>

<http://tiphero.com/wp-content/uploads/2017/05/pineapples-skin.jpg>

<https://thumbs.dreamstime.com/b/broccoli-texture-12426824.jpg>

<https://png.pngtree.com/thumb_back/fh260/back_pic/04/17/62/3858280e7464ddd.jpg>

<https://cdn.xl.thumbs.canstockphoto.com/boa-constrictor-snake-skin-stock-photograph_csp28251093.jpg>

<https://upload.wikimedia.org/wikipedia/commons/thumb/9/9e/Mamba_Dendroaspis_angusticeps.jpg/1200px-Mamba_Dendroaspis_angusticeps.jpg>

https://kids.nationalgeographic.com/content/dam/kids/photos/animals/Mammals/Q-Z/spotted-hyena-sideview.ngsversion.1396530777288.adapt.1900.1.jpg

https://www.asianscientist.com/wp-content/uploads/bfi_thumb/Bee-mite-genome-33dmy1u8zvnhptcp42mtc0.jpg

https://www.geneve.com/-/media/geneva/images/business/media/topics/bison-made-in-geneva/bison-made-in-geneva_1280x960.jpg

http://lemerg.com/887342.html

http://weknowyourdreams.com/single/gazelle/gazelle-05

https://assetsnffrgf-a.akamaihd.net/assets/m/102012369/univ/art/102012369_univ_lsr_xl.jpg

https://cdn.shopify.com/s/files/1/1625/3363/products/wg30_tmwgsm_1.jpg?v=1506670309

https://www.mileiq.com/blog/wp-content/uploads/2016/01/Odometer_70000-e1483039157734.jpg

https://news.nationalgeographic.com/content/dam/news/2016/09/03/WAQ_birds/01_waq_big_birds.jpg

http://www.sciencefocus.com/sites/default/files/iStock_61948836_XLARGE.jpg

https://upload.wikimedia.org/wikipedia/commons/thumb/7/72/American_flamingo_%28Phoenicopterus_ruber%29.JPG/1200px-American_flamingo_%28Phoenicopterus_ruber%29.JPG

http://www.wwf.org.au/Images/UserUploadedImages/415/img-dugong-swimming-in-sea-1400x600.jpg

https://i.imgur.com/TY7E85q.png

https://www.shutterstock.com/image-photo/basketball-isolated-on-white-background-135224051?irgwc=1&utm_medium=Affiliate&utm_campaign=TinEye&utm_source=77643&utm_term=

p10-schilling-maiko-a-20140918.jpg

jacquard-diamond-sock-hg08012-844.jpg

35891018-images-of-ice-cream.jpg

https://www.shutterstock.com/image-photo/closeup-macro-wild-african-rock-python-107571674?irgwc=1&utm_medium=Affiliate&utm_campaign=TinEye&utm_source=77643&utm_term=

armadillo1.jpg

http://www.mediabakery.com/PDI0166870-Wolves-Canis-lupus-nuzzling-in-snow-side-view.html

http://www.mediabakery.com/MNT0002049-Zebra-Equus-quagga-Masai-Mara-Reserve-Kenya.html

img-portrait-sumatran-tiger1400px.jpg

https://i.pinimg.com/736x/1b/af/f7/1baff727926487be1142b80c55b32459.jpg

**Glass Figurines**

Goose

<https://img0.etsystatic.com/163/0/5171596/il_340x270.1080231324_e1e4.jpg>

Peacock

<https://img.goingm.com/Images/DaleTiffany/AS14060.jpg>

Otter

<https://images-na.ssl-images-amazon.com/images/I/61vcP2FcQwL._SL1000_.jpg>

Elephant

<https://images.replacements.com/images/images2/crystal/I/P0000321198S0318T2.jpg>

Fox

<https://images-na.ssl-images-amazon.com/images/I/71SAmAQ-7wL._SY355_.jpg>

Tiger

<http://s651.photobucket.com/user/prochaska1/media/Tiger/Tiger6.jpg.html>

Shark

<https://img1.etsystatic.com/122/0/10103279/il_340x270.1103176363_4z1z.jpg>

Schooner

<https://i.pinimg.com/736x/e5/05/b5/e505b551894f66329c51cd745fbd650b--seas-sailing.jpg>

Totem pole

<https://img0.etsystatic.com/171/0/7458890/il_570xN.1120561660_sytp.jpg>

Bicycle

<https://i.pinimg.com/736x/cd/38/4b/cd384b10685ccc8dfa3c0f2b85199b5b--swarovski-crystal-figurines-swarovski-crystals.jpg>

Pineapple

<https://i.pinimg.com/originals/73/c1/71/73c1715af31599de9543f777953836ad.jpg>

Violin

<https://cdn3.volusion.com/vtaer.napck/v/vspfiles/photos/T1160-54114-2.jpg?1349261572>

Church

<http://shanfields.net/image/cache/data/figurines/waterford%20church-500x500.jpg>

Cannon

<http://static.tvtropes.org/pmwiki/pub/images/glass_cannon_by_swarleyswazenoskie-d604r4t_6338.png>

Piano

<https://images-na.ssl-images-amazon.com/images/I/41zxSi%2Bc%2BJL.jpg>

Umbrella

<https://blog.cmog.org/wp-content/uploads/2017/06/umbrella.jpeg>

Polar bear

<https://cdn0.rubylane.com/_pod/item/231976/3567/Chalet-Canada-Signed-Art-Glass-Polar-full-2-720-61.jpg>

**Outlines**

<http://moziru.com/images/jack-rabbit-clipart-black-and-white-16.jpg>

<http://www.publicdomainpictures.net/pictures/60000/velka/tiger-silhouette-1376081379LeR.jpg>

<http://weclipart.com/gimg/A275F518B9D4D3F4/polar-bear-silhouette-clip-art_29983.png>

<http://moziru.com/images/zebra-clipart-shadow-9.png>

<http://www.stickthisgraphics.com/images/Ibex%20Silhouette%20(Small).jpg>

<http://weclipart.com/gimg/5BDFD68F43B74913/il_340x270.558732768_jgjv.jpg>

<http://i.imgur.com/bSqxTxE.png>

<https://openclipart.org/image/2400px/svg_to_png/230426/snail_silhouette.png>

<https://www.polyvore.com/cgi/img-thing?.out=jpg&size=l&tid=106784033>

<http://www.clker.com/cliparts/0/w/n/S/q/6/black-bowtie-hi.png>

<http://cleanwipes.co.uk/wp-content/uploads/2015/09/fb-cup.jpg>

<http://www.supercoloring.com/sites/default/files/silhouettes/2015/05/lawn-mower-black-silhouette.svg>

<https://openclipart.org/image/2400px/svg_to_png/262794/RevolverSilhouette.png>

banana-outline-md.png

https://i.pinimg.com/originals/36/51/cf/3651cfe3d4f42047354230ad0656f6b2.gif

https://i.pinimg.com/originals/53/2e/b0/532eb042f99b869ac5ca508f1a41dab7.gif

https://tse3.mm.bing.net/th?id=OIP.g02Ww3ALidZiQx8ISWOfbgEsDU&pid=Api&w=255&h=181

http://www.clker.com/cliparts/i/Y/s/E/U/5/blue-outline-hammer.svg

http://www.downloadclipart.net/browse/12843/penguin-outline-clipart

https://images.clipartof.com/small/1064428-Grand-Piano-In-Black-And-White.jpg

https://i.pinimg.com/236x/af/64/bb/af64bba95ae1c2359dc3cdc223fa26d3--manta-ray-craft-manta-ray-tattoo.jpg

https://img1.etsystatic.com/050/0/5396130/il_340x270.721647499_otdk.jpg

http://www.supercoloring.com/sites/default/files/silhouettes/2015/05/wolf-outline-silhouette.svg

<http://clipart-library.com/clipart/982611.htm>

<https://i.pinimg.com/564x/c4/aa/52/c4aa529c56f4367d0f1622749da88392.jpg>

<http://www.vancitymommyd.com/wp-content/uploads/2018/01/butterfly-image-outline-butterfly-outline-free-download-clip-art-free-clip-art-on-dinosaur-clipart.jpg>

<https://i.pinimg.com/236x/56/be/7c/56be7c5f11b2e605b0998a3bf194c673--april-preschool-preschool-classroom.jpg>

<http://moziru.com/images/llama-clipart-simple-3.jpg>

<http://wecoloringpage.com/wp-content/uploads/2017/05/Airplane-Outline-Silhouette-Coloring-Page.jpg>

<http://moziru.com/images/drawn-teapot-template-11.gif>

<https://i.pinimg.com/originals/d8/62/01/d8620115af780afe330ac441c0a27e35.gif>

<http://www.infovia.net/cdn/6/2003/272/free-printable-pirate-ship-template_114689.gif>

<https://i.pinimg.com/originals/8b/3c/67/8b3c67c9907d04768744f2c428f17d7e.jpg>

**Experiment 5 Silhouettes**

http://clipart-work.net/data_gallery/animal-silhouette-silhouette-clip-art-and-silhouette-graphics-of-SVV2su-clipart.jpeg

http://clipart-library.com/hammer-images.html

vintage-mic-silhouette-clipart-of-black-19.jpg

http://silhouettegarden.com/files/images/football-jersey-silhouette.png

il_340x270.975845102_71er.jpg

1358029353524-704484808.jpeg
